# Stimulation of natural killer cells with small molecule inhibitors of CD38 for the treatment of neuroblastoma†

**DOI:** 10.1039/d2sc05749b

**Published:** 2023-01-30

**Authors:** Catherine M. Mills, Thomas Z. Benton, Ivett Piña, Megan J. Francis, Leticia Reyes, Nathan G. Dolloff, Yuri K. Peterson, Patrick M. Woster

**Affiliations:** a Department of Drug Discovery and Biomedical Sciences, Medical University of South Carolina 70 President St Charleston SC 29425 USA woster@musc.edu; b Department of Cell and Molecular Pharmacology and Experimental Therapeutics, Medical University of South Carolina 70 President St Charleston SC 29425 USA

## Abstract

High-risk neuroblastoma (NB) accounts for 15% of all pediatric cancer deaths. Refractory disease for high-risk NB patients is attributed to chemotherapy resistance and immunotherapy failure. The poor prognosis for high-risk NB patients demonstrates an unmet medical need for the development of new, more efficacious therapeutics. CD38 is an immunomodulating protein that is expressed constitutively on natural killer (NK) cells and other immune cells in the tumor microenvironment (TME). Furthermore, CD38 over expression is implicated in propagating an immunosuppressive milieu within the TME. Through virtual and physical screening, we have identified drug-like small molecule inhibitors of CD38 with low micromolar IC_50_ values. We have begun to explore structure activity relationships for CD38 inhibition through derivatization of our most effective hit molecule to develop a new compound with lead-like physicochemical properties and improved potency. We have demonstrated that our derivatized inhibitor, compound 2, elicits immunomodulatory effects in NK cells by increasing cell viability by 190 ± 36% in multiple donors and by significantly increasing interferon gamma. Additionally, we have illustrated that NK cells exhibited enhanced cytotoxicity toward NB cells (14% reduction of NB cells over 90 minutes) when given a combination treatment of our inhibitor and the immunocytokine ch14.18-IL2. Herein we describe the synthesis and biological evaluation of small molecule CD38 inhibitors and demonstrate their potential utility as a novel approach to NB immunotherapy. These compounds represent the first examples of small molecules that stimulate immune function for the treatment of cancer.

## Abstract 2

Small molecule inhibitors of CD38 promote increases in interferon gamma and stimulate natural killer cell proliferation for the treatment of neuroblastoma.

## Graphical abstract

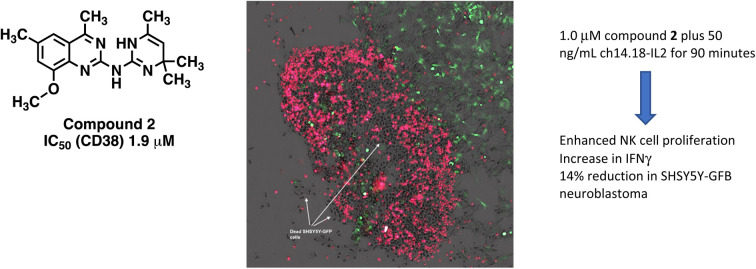

## Introduction

Neuroblastoma (NB) is a pediatric malignancy that occurs during fetal or early postnatal development. It is the most frequently diagnosed pediatric cancer during infancy and accounts for 15% of all pediatric cancer deaths.^1^ Nearly half of all NB patients will be classified as having high-risk disease, which is therapeutically challenging and has a poor prognosis.^2^ The advent of anti-ganglioside 2 (GD2) chimeric monoclonal antibody (mAb) immunotherapy for high-risk NB has improved 5 year survival for patients, but overall survival remains unacceptably low at under 50%.^3,4^ In high-risk NB patients, anti-GD2 mAbs such as naxitamab induce antibody-dependent cell-mediated cytotoxicity (ADCC). However, the overall success of anti-GD2 mAb immunotherapy for NB is highly dependent on the antitumor activity of natural killer (NK) and other effector cells,^5,6^ and failure to respond to treatment can be attributed to NB cell resistance^7^ or the inability of effector cells to kill tumor cells.^8,9^ As a result, the long-term efficacy of anti-GD2 mAb immunotherapy is unverified, and there is a pressing need for novel strategies to overcome resistance.^7^

It has been established that high-risk NB patients with increased RNA signatures for activated NK cells and CD8^+^ T cells experience improved outcomes.^10^ This implies that agents that prevent down regulation of immune function in the tumor microenvironment could represent a strategy for overcoming resistance to immunotherapy in NB and other cancers. The ectoenzyme cluster of differentiation 38 (CD38) has emerged as a potential target for immunomodulation^11^ and the anti-CD38 immune checkpoint inhibitors daratumumab and isatuximab have been approved for use in diffuse large B cell lymphoma, follicular lymphoma, mantle cell lymphoma and multiple myeloma (MM).^12,13^ CD38 is notable for its eccentric expression pattern, with predominant expression occurring in early and late stage T and B cell lymphocyte maturation.^14^ In addition to T, B, and myeloid cells, CD38 has been found to be constitutively expressed in NK cells,^15,16^ and is also a prognostic factor for multiple cancer types.^17^ In addition, CD38 plays a critical role in the homeostatic regulation of cellular energetics.^18,19^ By metabolizing the cofactor NAD^+^, CD38 removes an essential electron acceptor,^20^ thus limiting the energetic capacity of a cell. Importantly, CD38 over expression in immune cells and tumor cells within the tumor microenvironment (TME) causes a reduction in NAD^+^ levels, leading to down regulation of the immune response against tumor cells.^21^

**Fig. 1:**
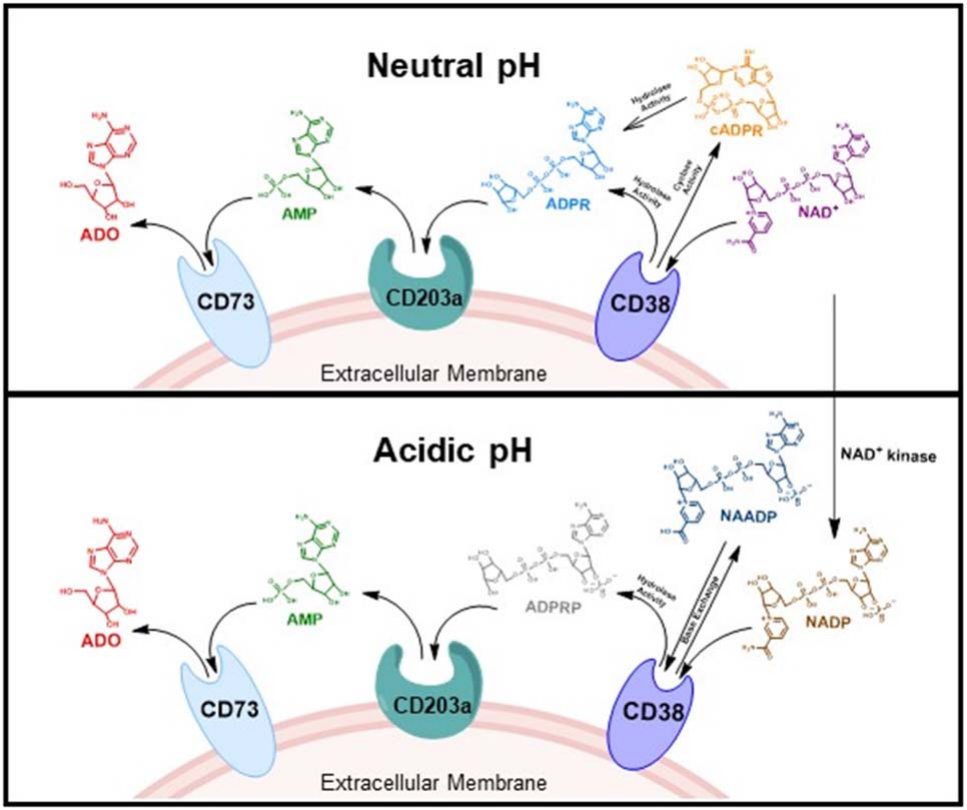
CD38-mediated extracellular ADO generation under neutral and acidic conditions. At neutral pH, CD38 hydrolyzes NAD^+^ or cADPR to ADPR. CD203a then hydrolyzes ADPR to AMP followed by CD73-mediated conversion of AMP to ADO. At acidic pH in the TME, CD38 converts NADP to NAADP *via* a base exchange reaction. NAADP is then hydrolyzed by CD38 to ADPRP which is converted to AMP and ADO by CD203a and CD73, respectively.

**Fig. 2:**
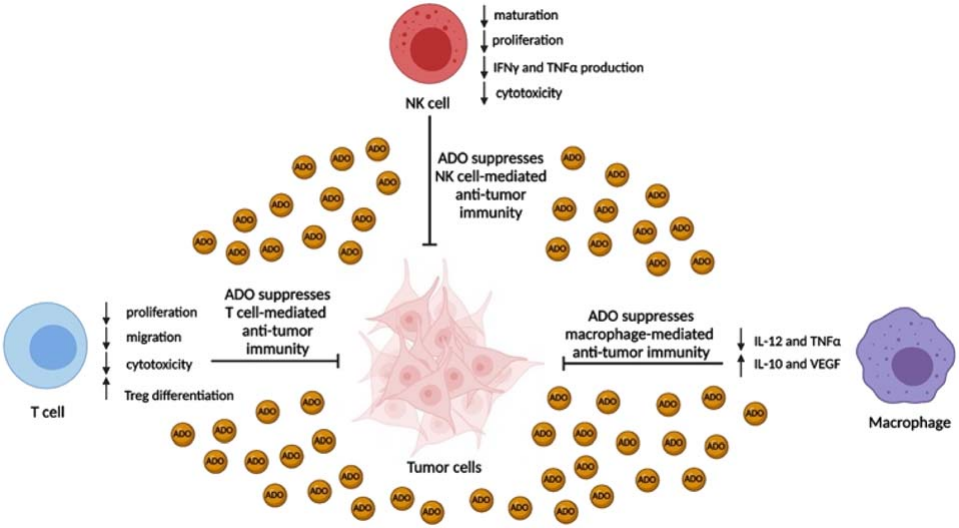
Immunosuppressive effects of ADO on immune effector cells. ADO concentration in the TME can often exceed 100 μM and ablate the anti-tumor activity of NK cells, T cells, and macrophages.

CD38 is a multifunctional enzyme, exhibiting both hydrolase and cyclase enzymatic activities, and regulates both a dominant and an alternative adenosine (ADO) pathway (Fig. 1).^20,22^ The better-known pathway involves the nucleoside triphosphate diphosphohydrolase known as cluster of differentiation 39 (CD39).^22^ The optimal pH for CD39-mediated hydrolysis of ATP and ADP is 8.0–8.5, and in normal tissue the CD39 pathway is the predominant source of exogenous ADO. However, it has been suggested that ADO production *via* CD38 ^22,23^ (Fig. 1) likely predominates in the acidic TME.^24–27^ ADO is considered a crucial mediator of the immune response (Fig. 2), and ADO receptors are known to be expressed in various immune cells, where they mediate the regulation of immune and inflammatory responses.^28^ Extracellular ADO, which is prominent in the TME, stimulates ADO receptor subtype 2A, (A_2A_AR)^29,30^ on immune cells, including T cells, natural killer cells, neutrophils, macrophages and dendritic cells, preventing their activation and driving naïve CD4^+^ T cell differentiation toward a Treg immunosuppressive phenotype.^31,32^ In NK cells, ADO suppresses their cytotoxic activity toward tumor cells and their production of IFN-γ, tumor necrosis factor (TNF-α) and granulocyte-macrophage colony-stimulating factor (GM-CSF), which are critical cytokines for effective ADCC.^29,30,33,34^ In tumor cells that highly express CD38, such as MM,^35–37^ NB^38–40^ and acute lymphocytic leukemia (ALL),^41,42^ both reduction in NAD^+^ levels and overproduction of ADO lead to immunosuppression. Recent evidence suggests that CD38 up regulation is one of the most important factors in mediating resistance to checkpoint blockade in MM and other cancers.^43–45^ In addition, resistance to PD-1/PD-L1 blocking antibodies is mediated through up regulation of CD38 and subsequent production of ADO.^43^ Numerous small molecule NAD^+^ mimetics have been developed and tested for CD38 inhibition in the context of aging, mitochondria dysfunction, obesity, and diabetes.^18,19,46–48^ Notably, some of these inhibitors were successful in enzymatic studies, and in some cases promoted increases in the levels of NAD^+^*in vivo*, but none were evaluated for antitumor or immunostimulatory effects.^19,46,48,49^ Our group recently reported that enzymatic inhibition of CD38 hydrolase or cyclase activity in activated human peripheral blood mononuclear cells (PBMCs) resulted in an 82% increase in cellular NAD^+^ and a >100-fold increase in interferon gamma (IFN-γ) secretion.^20^ We now report the design and synthesis of a limited series of quinazoline-dihydropyrimidine-based CD38 inhibitors related to compound, 1 ^20,50,51^ (Compounds 2–13, Table 1) and describe their immunostimulatory and pro-proliferative effects on NK cells. These analogues can be used to enhance the cytotoxic effect of NK cells toward NB cells *in vitro*.

**Table 1:** Structures of CD38 inhibitors and their activity against CD38 hydrolase. Compound identifier, compound structure, % CD38 hydrolase remaining activity at 50.0 μM compound, IC_50_, and Tanimoto coefficient data for select compounds. ND = not determined

Cmpd	Structure	% CD38 hydrolase activity remaining (50.0 μM)	IC_50_ hydrolase activity (μM)	Tanimoto coefficient
1	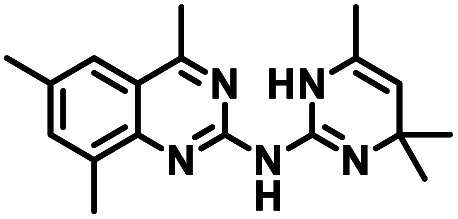	61.6 ± 2.0	4.2 ± 0.5	Reference compound
2	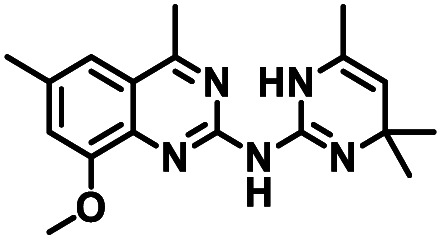	5.6 ± 0.8 (25 μM)	1.9 ± 0.1	0.880
3	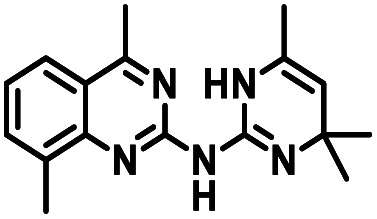	41.0 ± 0.03	8.4 ± 0.7	0.957
4	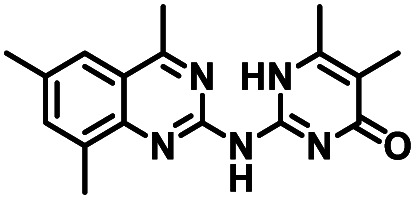	58.2 ± 1.4	5.1 ± 0.9	0.484
5	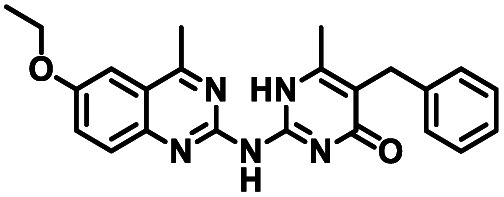	57.0 ± 0.3	8.3 ± 1.9	0.325
6	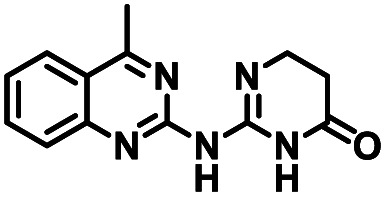	66.8 ± 0.6	ND	0.680
7	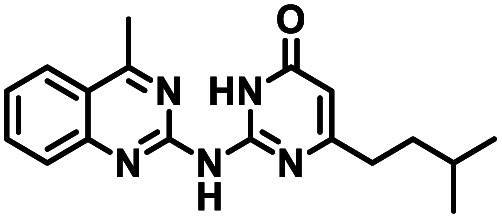	60.3 ± 0.7	4.0 ± 0.9	0.382
8	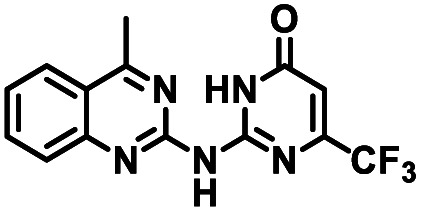	53.2 ± 0.2	20.6 ± 3.3	0.394
9	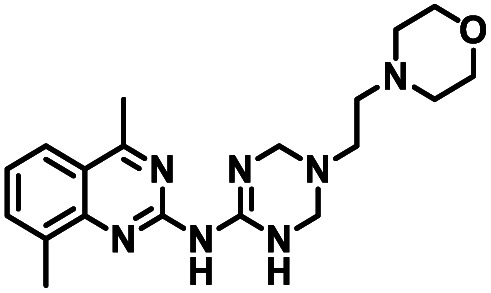	65.3 ± 0.6 (25 μM)	ND	0.563
10	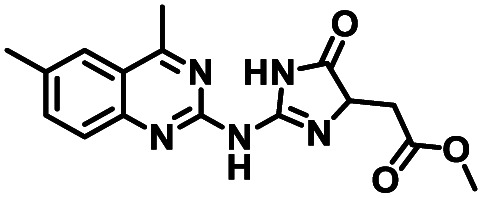	61.4 ± 3.8	11.9 ± 2.6	0.621
11	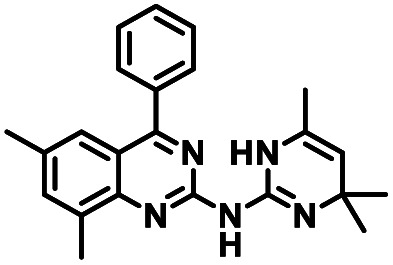	49.1 ± 2.2	23.8 ± 1.8	0.821
12	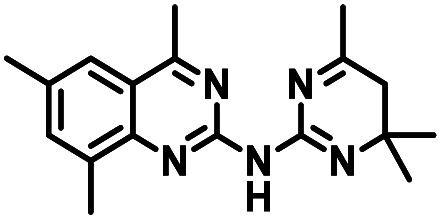	52.0 ± 9.6	10.8 ± 0.7	0.840
13	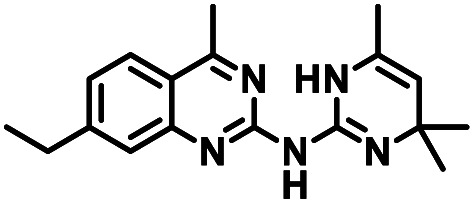	48.1 ± 6.8	22.4 ± 1.1	0.840
14	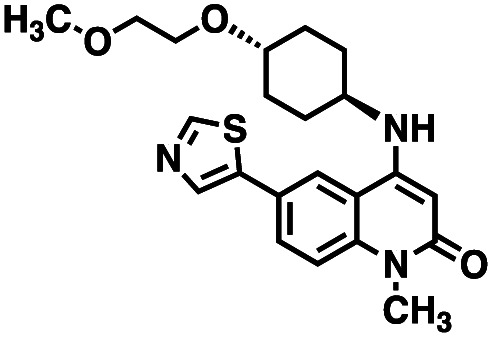	60.3 ± 1.8	0.078 ± 0.03	ND

## Results

### Correlation of CD38/CD73 expression in NB

**Fig. 3:**
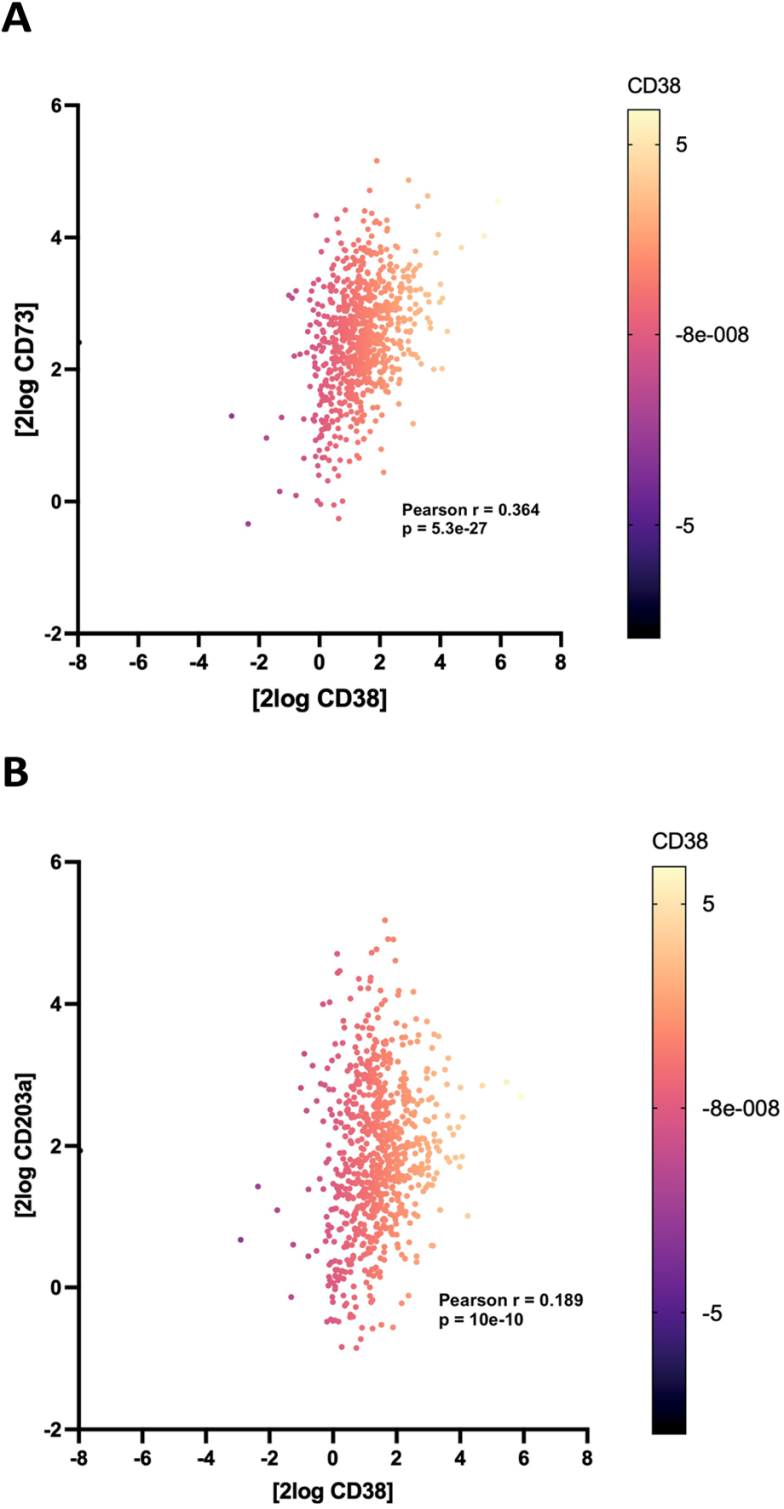
Correlation between the expression of CD38 and CD73 (panel A) and CD38 and CD203a (panel B) from *in silico* analysis of 786 neuroblastoma patients in the Cangelosi database. Lighter colors in the heat maps indicate higher expression levels.

To support the contention that the CD38 adenosinergic pathway was prevalent in neuroblastoma, we performed *in silico* analysis of CD38 and CD73 expression levels in neuroblastoma samples from 786 patients in the Cangelosi neuroblastoma database.^52^ As shown in Fig. 3, there is a strong correlation between expression of CD38 and CD73 (*p* = 5.3 × 10^−27^) and between CD38 and CD203a (*p* = 10 × 10^−8^), suggesting that this pathway is actively producing ADO, leading to immunosuppression. As indicated by the associated heat map, a large percentage of these samples had high expression of all three of these enzymes.

#### Preliminary structure activity relationship analysis

**Scheme 1:**
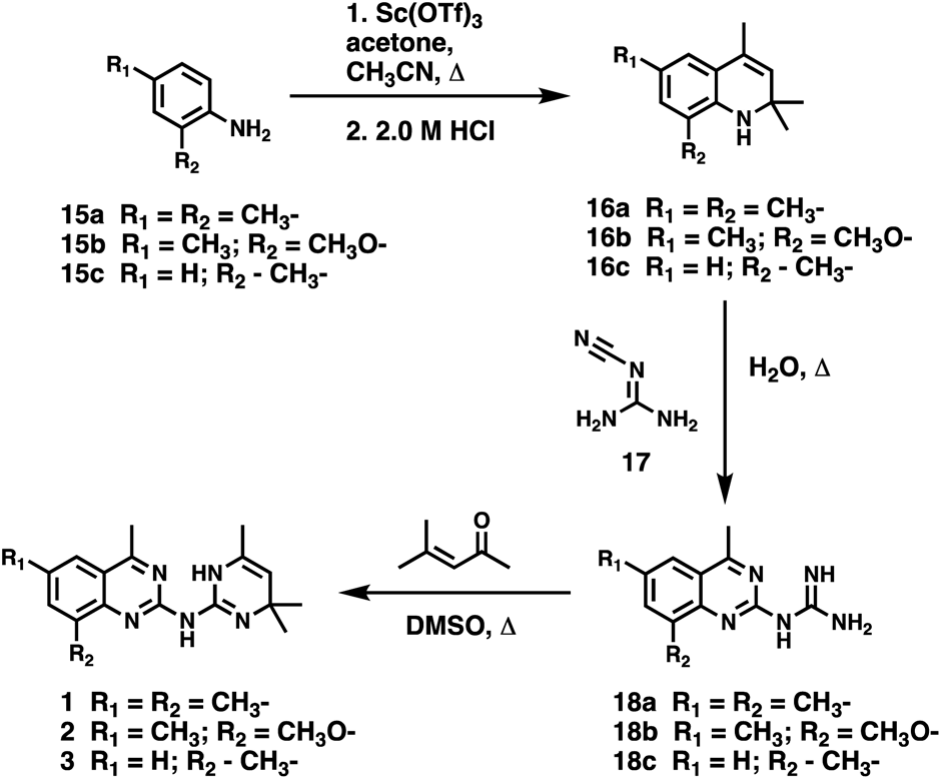


**Fig. 4:**
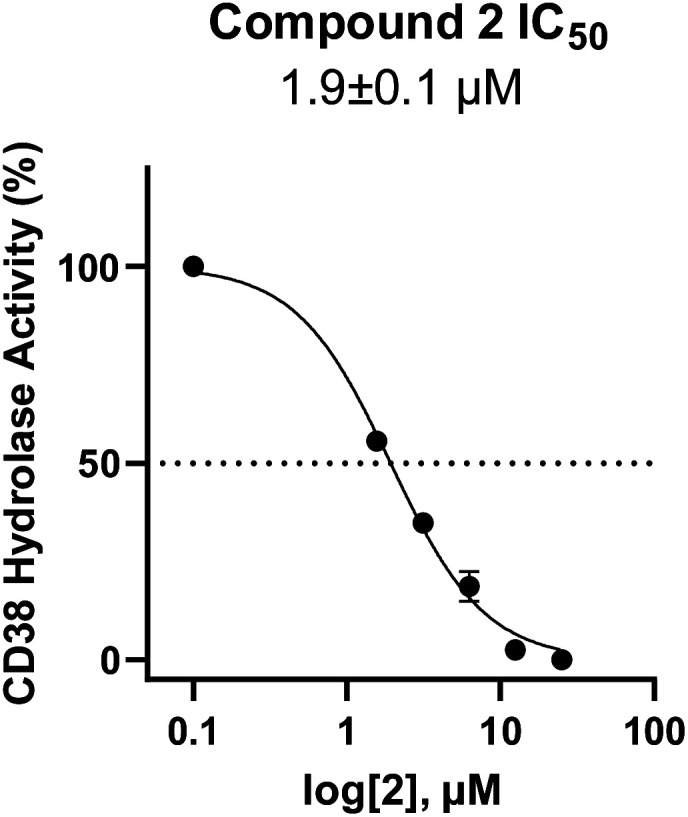
Inhibition of CD38 hydrolase activity by 2. CD38 hydrolase activity (%) *vs.* log [2] (μM). Each data point is the average of readings from 3 or more separate wells ± SEM.

To facilitate structural optimization of compound 1, a synthetic route to 1 and the previously unknown compounds 2 and 3 was completed in 3 steps, as shown in Scheme 1. A modified Skraup synthesis was used to convert 2,4-dimethylaniline 15a to the corresponding 1,2-dihydroquinoline 16a (Sc(OTf)3, acetonitrile, heat).^53^ Intermediate 16a was treated with 2-cyanoguanidine 17 to form the biguanide intermediate 18a,^54^ which was converted to 1 in the presence of 4-methylpent-3-en-2-one.^55^ A structure search in SciFinder® revealed 200 analogues related to 1, which were available in our in-house South Carolina Compound Library (SC^3^) or purchased (Vitas-M Laboratory, Hong Kong). These compounds were evaluated for CD38 hydrolase inhibition (data not shown) in our previously published assay.^20^ Compounds 2–13 (Table 1) proved to be the most potent inhibitors, and were evaluated for structural similarity using the Tanimoto method.^56^ The known CD38 inhibitor 14 was included as a positive control in all experiments at a concentration of 50.0 nM. Compounds 4 (IC_50_ = 5.1 ± 0.9 μM) and 7 (IC_50_ = 4.0 ± 0.9 μM) exhibited potencies comparable to hit compound 1 (IC_50_ = 4.0 ± 0.5 μM), indicating that derivatization of the dihydropyrimidine ring is tolerated, particularly at the 4- and 5-positions of the dihydropyrimidine ring. Interestingly, while alkyl-substitution at the 4-position of the dihydropyrimidine ring was permitted without impacting activity, addition of an electron withdrawing trifluoromethyl group in 8 (IC_50_ = 20 ± 3 μM) reduced potency 5-fold. Compound 3 (IC_50_ = 8.4 ± 0.7 μM) exhibited a 2-fold decrease in potency, suggesting that substitution at the 6-position of the quinazoline ring is an important chemical feature. Ethoxy substituents at the 6-position of the quinazoline ring and bulky benzyl substitution at the 5-position of the dihydropyrimidine ring, as seen in 5 (IC_50_ = 8 ± 2 μM), did not diminish activity significantly, resulting in only a 2-fold decrease in potency. Changing the conjugation of the dihydropyrimidine ring in 12 (IC_50_ = 10.8 ± 0.7 μM) appeared to decrease potency nearly 3-fold. Addition of a bulky phenyl group at the 4-position of the quinazoline ring, as seen in 11 (IC_50_ = 23 ± 1 μM), reduced activity 5-fold. Simple replacement of the methyl group at the 8-position of the quinazoline ring with a methoxy group in 2 (IC_50_ = 1.9 ± 0.1 μM, Fig. 4) resulted in a 2-fold increase in potency, indicating that modifications at the 8-position could lead to enhanced activity. Finally, replacement of the dihydropyrimidine ring with the dihydroimidazole as in 10 (IC_50_ = 12 ± 2 μM) resulted in a 3-fold decrease in potency, suggesting that while the dihydropyrimidine ring is an important scaffold component, there is room for modification without eliminating activity entirely.

#### *In silico* studies

**Fig. 5:**
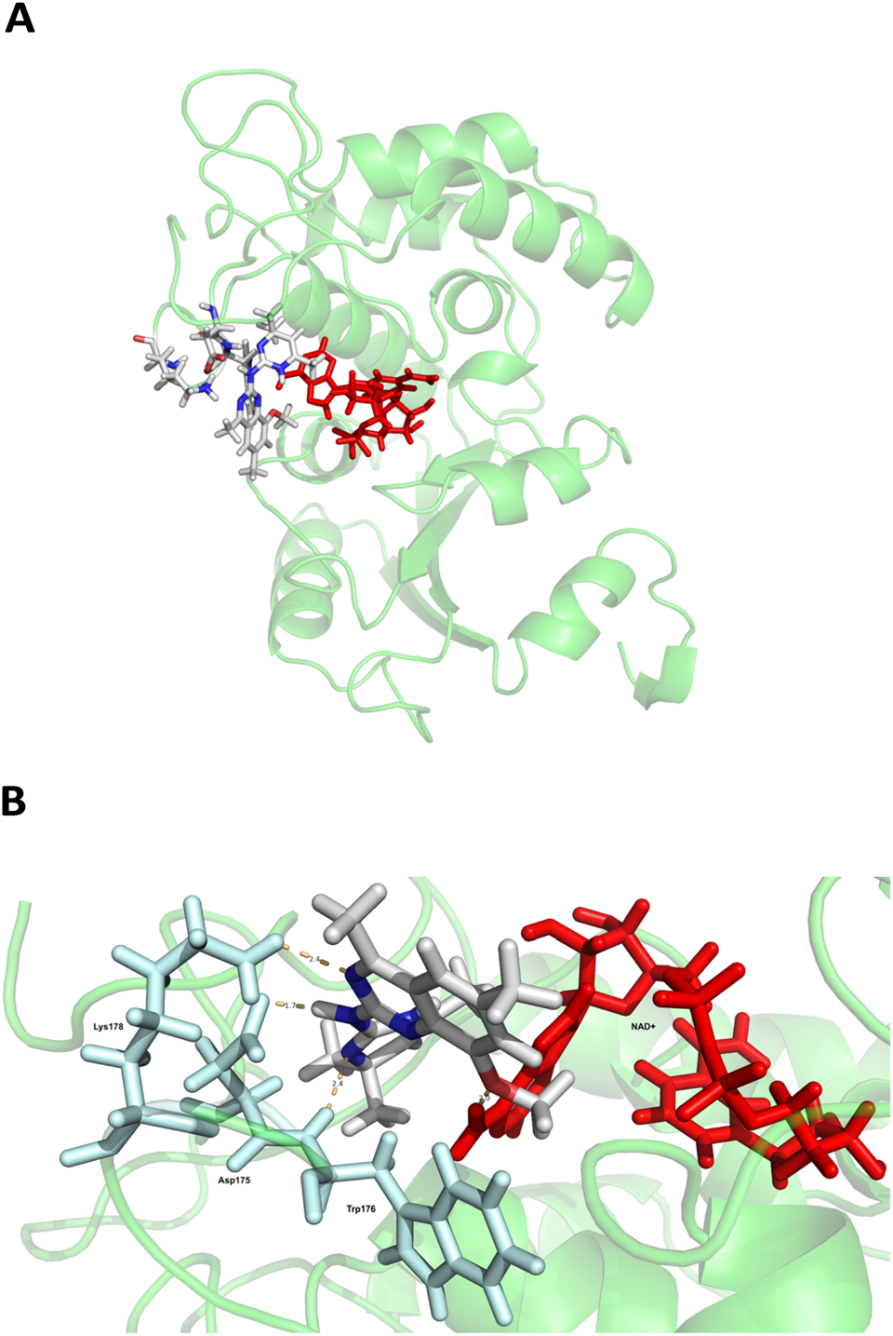
Compound 2 docked with CD38^(E226)^-NAD^+^ complex. Panel A: compound 2 docked in the active site of CD38^(E226)^-NAD^+^ complex, panel B: interactions of compound 2 with Asp175, Trp176, Lys178 and NAD^+^ (amino acids shown as cyan sticks, NAD^+^ shown as red sticks).

We next created a model of the potential interactions and bonding of 2 with CD38 at the molecular level through *in silico* docking and molecular dynamics. Using molecular operating environment (MOE) Dock, compound 2 was docked with a CD38 X-ray crystal structure (CD38^E226^, RCB PDB: 2I66, Fig. 5A and B).^57^ Docking parameters were set to allow the receptor and ligand to flex, and to predict enzyme/ligand affinity. Molecular dynamics were run as described in the Experimental section, revealing several potentially required interactions. The secondary amine at the 2-position of the dihydroquinazoline ring of 2 appears to form a 1.7 Å hydrogen bond with Asp175. Likewise, the nitrogen at the 3-position of the dihydroquinazoline ring forms a 2.4 Å hydrogen bond with Lys178, the sp^2^ nitrogen alpha to the gem dimethyl of the dihydropyrimidine ring hydrogen bonds to Trp176 (2.4 Å) and the dihydroquinazoline methoxy oxygen bonds to NAD^+^ (2.0 Å). There also appear to be pi–pi interactions between the dihydroquinazoline aromatic ring and the adenine ring of NAD^+^ (not shown). These data will be useful to facilitate the structure-based optimization of 2.

#### Effect of CD38 hydrolase inhibition in peripheral blood NK cells

**Fig. 6:**
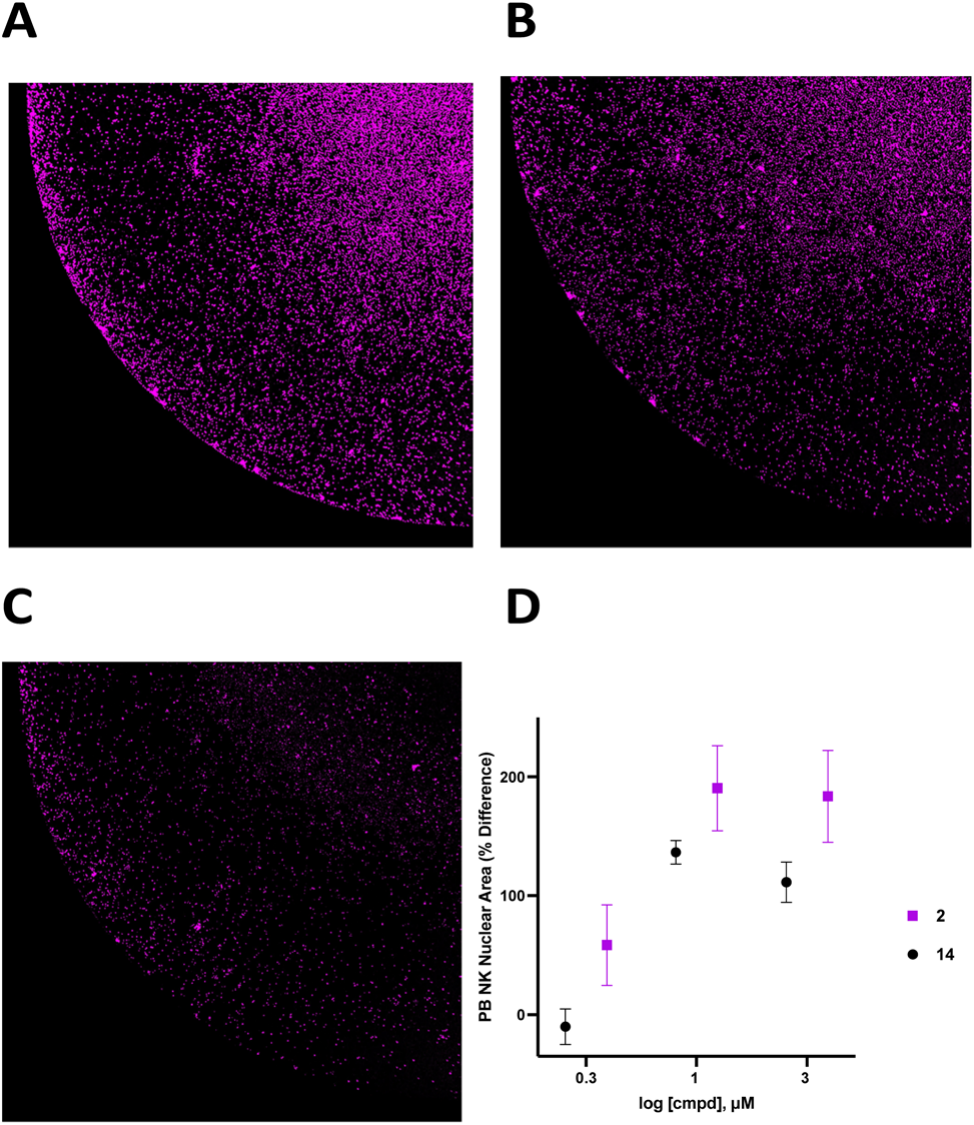
Images of PB NK cells following 48-treatment and cell quantitation. Panel A: image of PB NK cells following 48 hour treatment with 1 μM compound 2. Cells were stained with Hoechst and imaged at 4× magnification using a BioTek Cytation 5 imager. Panel B. Image of PB NK cells following 48 hour treatment with 1 μM compound 14. Cells were stained with Hoechst and imaged at 4× magnification using a BioTek Cytation 5 imager. Panel C: image of PB NK cells following 48-treatment with vehicle control. Cells were stained with Hoechst and imaged at 4× magnification using a BioTek Cytation 5 imager. Panel D: PB NK nuclear area (% difference) at 0.3, 1, and 3 μM compound 2. Each data point is the are the average of readings from 6 or more separate wells (3 from each donor) ± SEM. Data values in panel D are normalized to vehicle.

Human peripheral blood (PB) NK cells (StemCell Technologies, Vancouver, CA, purity > 90%) were transferred to a 96-well plate at a confluency of 40 000 cells per well in RPMI 1640 + 100 IU mL^−1^ IL-2 with or without 1.0 μM 2 for 48 hours, and live cells were stained with Hoechst (final concentration 1 μM) and imaged at 4× magnification using a BioTek Cytation 5 imager (Fig. 6A–C). Cells in each treatment group were normalized to vehicle control and data was analyzed as PB NK cell area % difference as a function of compound concentration (Fig. 6C). A dose–response relationship for 2 and 14 is shown in Fig. 6D. Treatment with 1.0 μM compound 2 produced a 190 ± 36% increase in nuclear area. Additionally, there was extensive clumping in cells treated with 2 relative to vehicle control. Cell clumping is characteristic of proliferating immune cells.^58,59^ These data suggest that treatment of NK cells with inhibitors of CD38 hydrolase experience an expansion-like effect similar to what is observed when NK cells are expanded with IL-2 and/or feeder cells.^59^

**Fig. 7:**
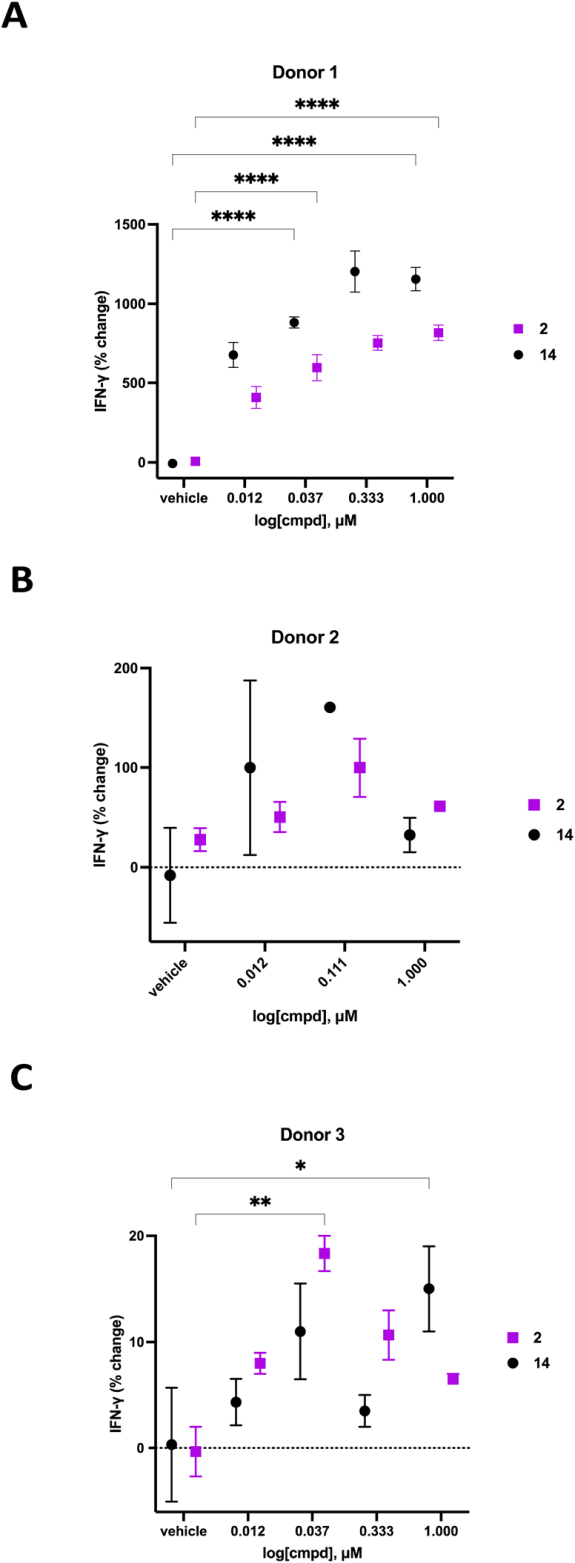
Effect of 24 hour exposure of 2 and 14 on PB NK cell IFNγ secretion. Panel A: percent change in IFNγ for donor 1 PB NK cells treated with 2 or 14, panel B: percent change in IFNγ for donor 2 PB NK cells treated with 2 or 14, panel C: percent change in IFNγ for donor 3 PB NK cells treated with 2 or 14. IFNγ was quantified using a Promega IFNγ lumit assay. Each data point is the average of readings from at least 3 separate wells ± SEM. Data analyzed by multiple comparison two-way ANOVA: *p ≤ 0.05, **p ≤ 0.01, ***p ≤ 0.001, ****p ≤ 0.0001.

We previously demonstrated that compounds related to 2 have the ability to increase cellular NAD^+^ and IFNγ levels in human PBMCs *in vitro*. In light of the substantial change in viability/proliferation of PB NK cells following treatment with 2, changes in the ability of treated NK cells to secrete IFNγ were measured.^60,61^ Human PB NK cells (purity > 90%) were added to a 96-well plate at a confluency of 49 000 cells per well in Immunocult-XF T cell expansion medium supplemented with 500 IU mL^−1^ IL-2, 10 ng mL^−1^ IL-15, and 0.2 μL mL^−1^ CD2/CD3/CD28 T cell activator. These cells were exposed to varying concentrations of 2 or 14 for 24 hours, followed by quantification of IFNγ using a Lumit IFNγ assay kit (W6040, Promega, Madison, WI). Treatment groups were normalized to vehicle-treated control and data was expressed as percent change in IFNγ. The resulting IFNγ data for donors 1–3 are presented in Fig. 7. As often happens, we encountered a wide variability in the response of human primary cells from different donors. Donor 1 exhibited the most robust dose-dependent increase in IFNγ production with an 759 ± 45% increase at 1.0 μM 2. Likewise, donor 2 exhibited a 100 ± 29% dose-dependent increase in IFNγ at 0.111 μM 2 while donor 3 exhibited an 18 ± 2% increase in IFNγ at 0.037 μM 2. While the amplitude of the change in IFNγ varied significantly from donor to donor, a concentration-dependent response was observed in all donors treated with 2. The known CD38 inhibitor 14 produced increases in IFNγ levels that were similaer to the effects of 2 in all 3 donors. Despite the variability in the magnitude of the IFNγ response, the trend in all 3 donors was a dose-dependent increase for both 2 and 14. Decreases in IFNγ expression at higher doses of both compounds in donors 2 and 3 may be due to modest cytotoxicity.

**Fig. 8:**
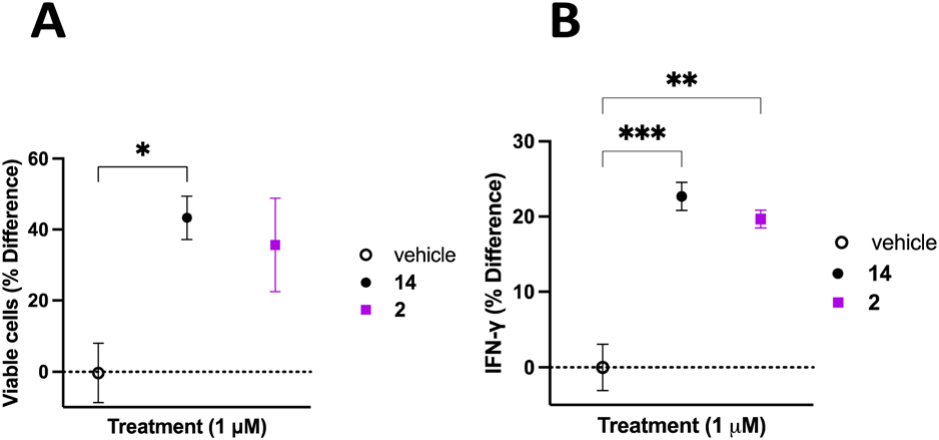
Effect of small molecule inhibitors of CD38 hydrolase activity on PB NK cells over an extended period. Panel A: percent difference in viable cells treated with vehicle, 14 or 2 every 3 days for 20 days. Each data point is the average of at least 3 independent determinations ± SEM, panel B: percent change in IFNγ for PB NK cells treated with vehicle, 14 or 2 every 3 days for 20 days. IFNγ was quantified using a Promega Lumit IFNγ assay. Each data point is the average of readings from at least 3 separate wells ± SEM. One-way ANOVA: *p ≤ 0.05, **p ≤ 0.01, ***p ≤ 0.001.

The TME has the ability to down regulate NK cell activation and function, therefore it is important to discern whether inhibition of CD38 hydrolase activity over an extended period will improve NK cell activation and support a persistent response.^62^ To address this question, donor 3 PB NK cells were plated on a 12-well plate at a confluency of 1 × 10^6^ cells mL^−1^ in Immunocult-XF T cell expansion medium supplemented with 500 IU mL^−1^ IL-2, 10 ng mL^−1^ IL-15, and 0.2 μL mL^−1^ CD2/CD3/CD28 T cell activator. Cells were treated with vehicle control, 2, or 14. Cells were treated with fresh medium every 3 days over the course of 20 days. Cells from each treatment group were counted on the 20^th^ day of treatment and viability determined using trypan blue. NK cells treated with 2 or 14 were normalized to vehicle control with the percent change in viable cells presented as a function of the treatment condition (Fig. 8A). We observed 43 ± 6% more viable cells in the group treated with 14 and 36 ± 13% more viable cells in the group treated with 2, relative to vehicle control. Additionally, on the 20^th^ day of treatment the cell supernatant was collected and analyzed for IFNγ using the Promega Lumit IFNγ assay. Treatment groups were normalized to vehicle control and the data presented as IFNγ % difference as a function of treatment (Fig. 8B). We observed 23 ± 2% and 20 ± 1% more IFNγ secreted by PB NK cells treated with 14 and 2, respectively, relative to vehicle control. Collectively, this data indicates that long-term treatment with inhibitors of CD38 hydrolase activity produce more viable cells, and subsequently more IFNγ, relative to vehicle treated control.

**Fig. 9:**
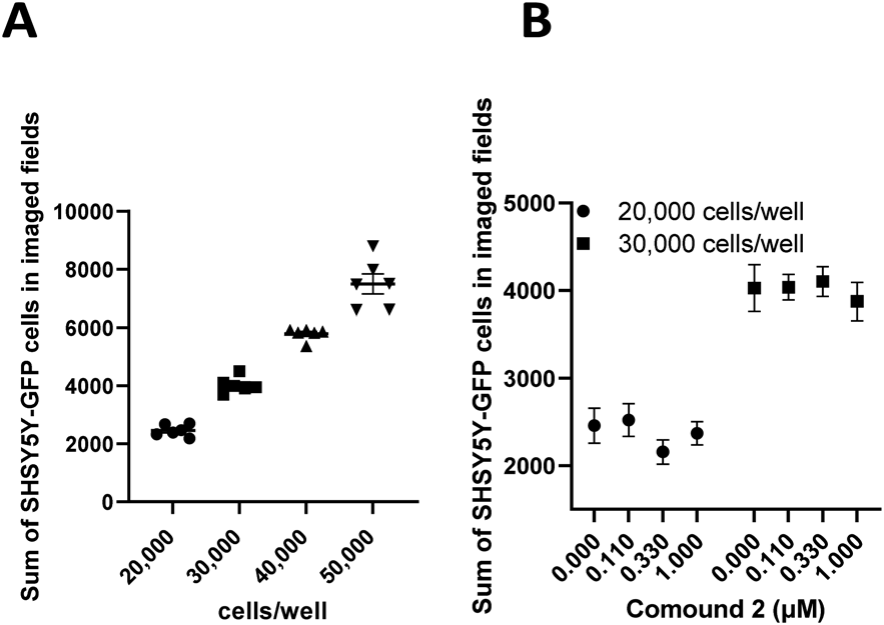
SHSY5Y-GFP cell plating optimization for co-culture experiment. Panel A: quantitation of SHSY5Y-GFP cells at different densities. Cells were imaged at 4× magnification using a BioTek Cytation 5 imager; panel B: SHSY5Y-GFP cells were plated at 20 000 cells per well or 30 000 cells per well and treated with different concentrations of 2. Cells were imaged at 20× using a BioTek Cytation 5. Each data point is the average of readings from at least 6 separate wells ± SEM.

**Fig. 10:**
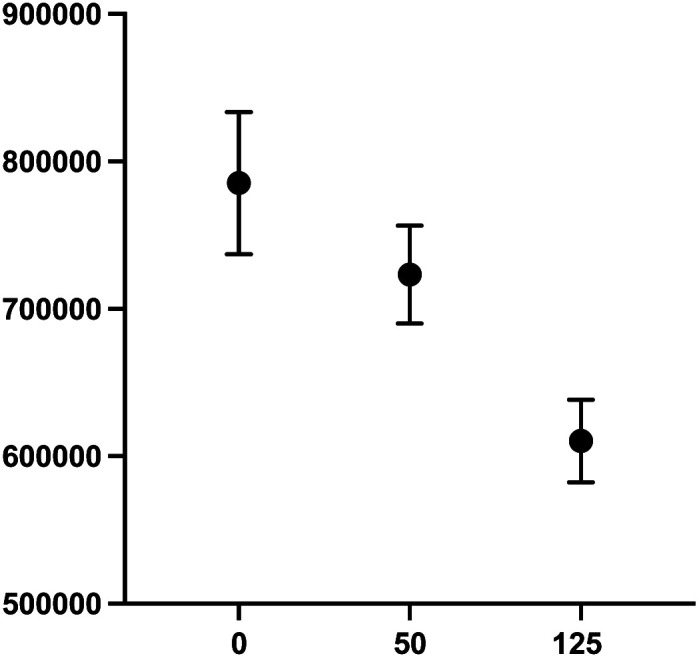
Optimization of ch14.18-IL2 concentration in SHSY5Y-GFP and PB NK cell co-culture. SHYS5Y-GFP and PB NK cells (at 25 000 cells per well and an effector cell : target cell ratio of 1 : 1). PB NK cells were stained with cell tracker deep red. Concentrations of 50 and 125 ng mL^−1^ ch14.18-IL2 were tested, with cells incubated for 90 minutes. Cells were imaged at 20× and the sum of GFP integrated fluorescence was measured using a BioTek Cytation 5 image. Each data point is the average of readings from 3 or more separate wells ± SEM.

Undifferentiated SHSY5Y NB cells grow in mounding clusters, which is potentially problematic for cell imaging.^63^ Therefore, prior to any co-culture experiments, we wanted to determine an optimal cell plating number per well. Since compound 2 had a significant effect on stimulation of NK cell proliferation, we used this compound to optimize the assay conditions. These experiments suggested that plating at 20 000–30 000 cells per well produced an optimal signal to noise ratio with more precision across all wells (Fig. 9). Again, compound 2 was employed to optimize assay conditions. As shown in Fig. 10, a concentration-dependent response with ch14.18-IL2 treatment was observed, and 50 ng mL^−1^ of ch14.18-IL2 was determined to be optimal.

Dinutuximab is the standard of care immunotherapy for high-risk NB treatment. The high cost and unavailability of dinutuximab made it unsuitable for our studies, therefore, we chose an alternative clinically relevant targeted biologic. The fusion protein ch14.18-IL2 contains a chimeric anti-GD2 antibody (ch14.18) tethered to recombinant human IL-2.^64^ The IL-2 portion of the immunocytokine activates NK cells *via* the IL-2 receptor instead of the Fcγ receptor as seen with dinutuximab.^65,66^ In addition, it has exhibited activity in NB-bearing mice *via* NK-mediated effects and enhanced antitumor activity when compared to anti-GD2 antibody in combination with IL-2.^67,68^ Thus we examined whether combined treatment with ch14.18-IL2 and an inhibitor of CD38 would enhance the cytotoxic effects of NK cells. To conserve PB NK cells, the concentration of ch14.18-IL2 was optimized to elicit a sufficient response using an effector cell : target cell ratio of 1 : 1.

**Fig. 11:**
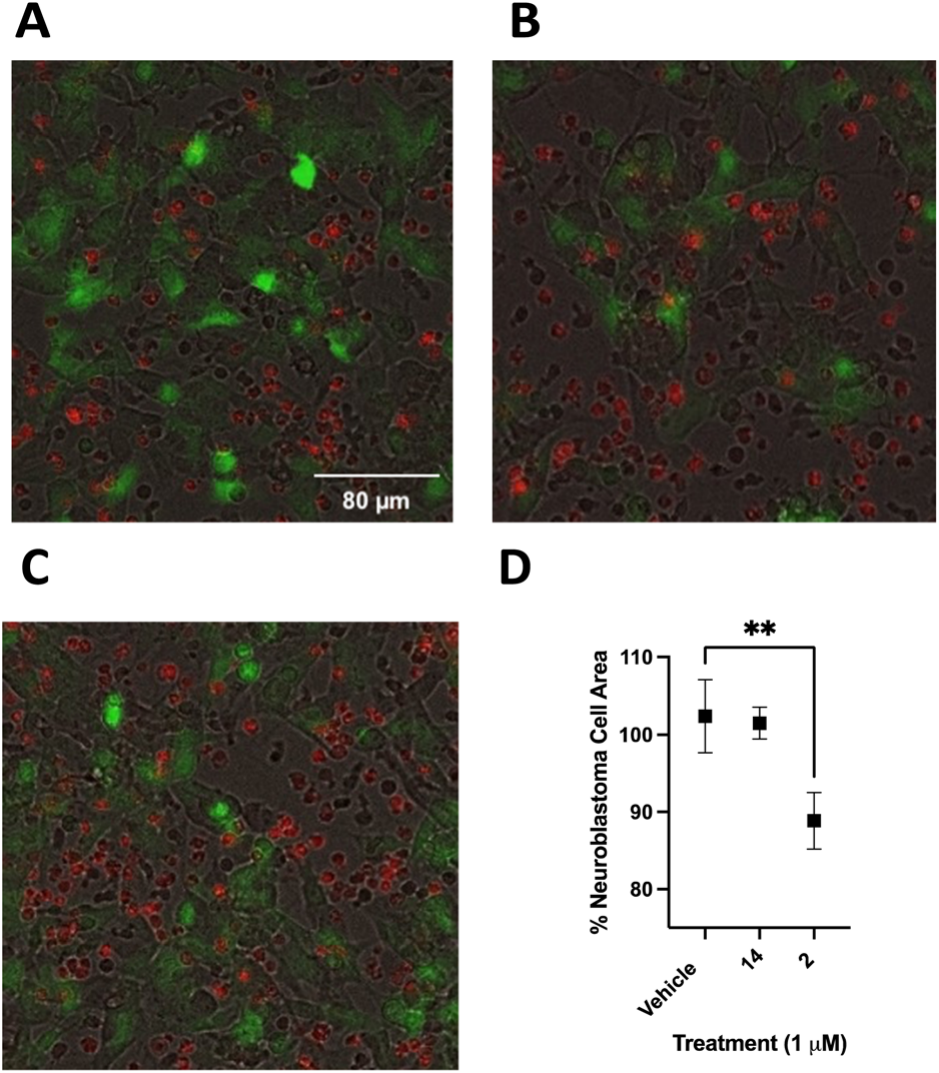
Cytotoxic effects of PB NK cells toward SHSY5Y-GFP cells with combination treatment of small molecule inhibitors of CD38 hydrolase activity and 50 ng mL^−1^ ch14.18-IL2. Panel A: SHSY5Y-GFP (green) and PB NK cells (red) in combination with 50 ng mL ch14.18-IL2 and vehicle, panel B: SHSY5Y-GFP (green) and PB NK cells (red) in combination with 50 ng mL ch14.18-IL2 and 2, panel C: SHSY5Y-GFP (green) and PB NK cells (red) in combination with 50 ng mL ch14.18-IL2 and 14. Cells were imaged at 20× with a BioTek Cytation 5. Panel D: the sum of SHSY5Y-GFP cells was quantified by the sum of integrated GFP fluorescence intensity and normalized to vehicle. Black bodies in each image are dead SHSY5Y-GFP cells. Each data point is the average of readings from 3 or more separate wells ± SEM. **p ≤ 0.01.

**Fig. 12:**
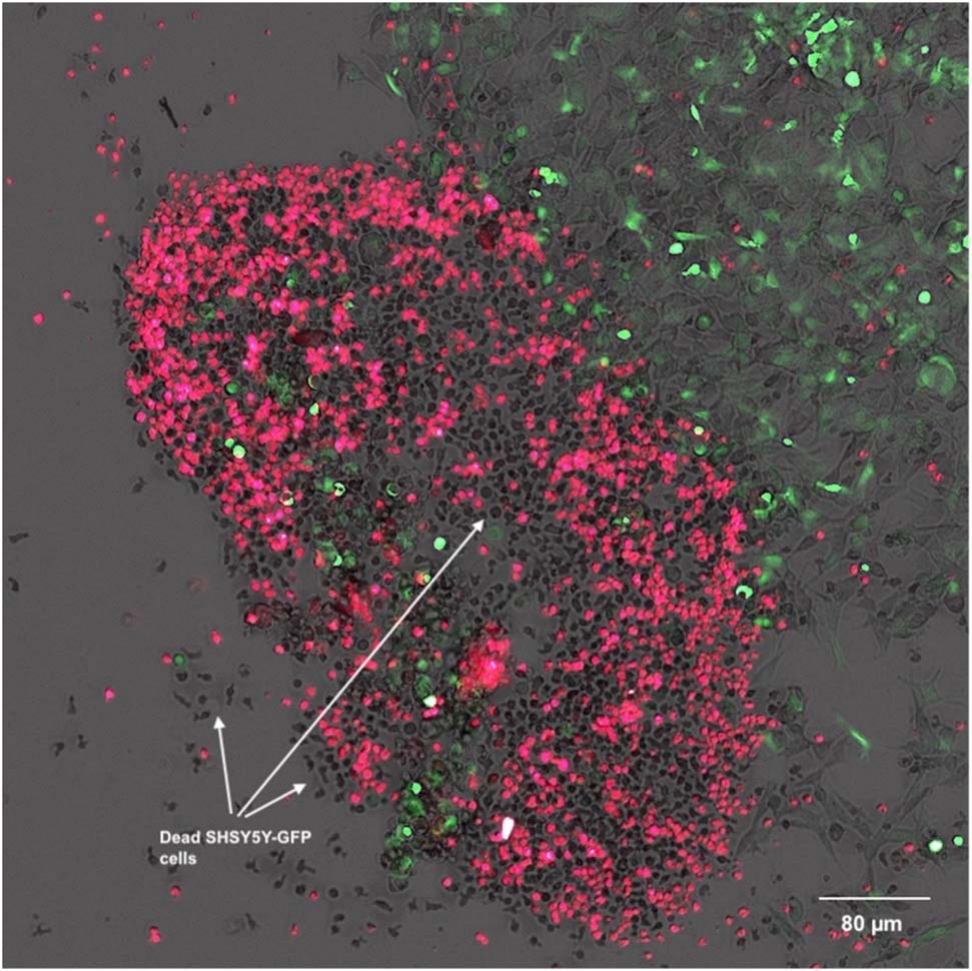
Effect on the response of natural killer (NK) cells of compound 2 against SH-SY5Y NB cells transfected with GFP. NK cells are stained red. Cells were treated with 50 ng mL^−1^ of recombinant ch14.18 IL-2 and 1 μM of each analogue for 90 minutes. The image shows NB cells (green) being destroyed by NK cells (red). Black bodies in the image are dead SHSY5Y-GFP cells. Images were produced on a Biotek Cytation 5 cell imager.

Following optimization, SHSY5Y-GFP cells were plated at 25 000 cells per well and incubated for 24 hours. PB NK cells stained with cell tracker deep red were added to SHSY5Y-GFP cells with 50 ng mL^−1^ ch14.18-IL2 and treated with 2, 14 or vehicle control. Cells were incubated for 90 minutes at 37 °C and imaged using a BioTek Cytation 5 at 20× (Fig. 11A–C). Live SHSY5Y-GFP cells were quantitated by measuring the integrated GFP fluorescence intensity. After only 90 minutes treatment with a 1.0 μM concentration of 2 and 50 ng mL^−1^ ch14.18-IL2a caused a 14 ± 3% decrease in SHSY5Y-GFP fluorescent area (Fig. 11B) relative to vehicle-treated control (Fig. 11A). Interestingly, there was not a statistically significant difference between cells treated with 1 μM 14 (Fig. 11C) and vehicle-treated control. These results are depicted graphically in Fig. 11D. Fig. 12 is a close-up view from a representative well showing the effects of 1 μM 2 combined with 50 ng mL^−1^ ch14.18-IL2.

## Discussion

CD38 up regulation is thought to be one of the most important factors in mediating resistance to checkpoint blockade in MM and other cancers.^43–45^ CD38-targeted biologics are currently used in the clinic to treat MM, but they do not mitigate the immunomodulating effects of CD38. The CD38-targeting biologic daratumumab has modest inhibitory activity against CD38 cyclase activity and enhances CD38 hydrolase activity.^69^ In addition, CD38 mAbs may also mask regions of the CD38 epitope that are necessary for important CD38 receptor functions that promote NK cell interferon secretion and tumor cell cytotoxicity. Thus CD38-targeted biologics like daratumumab would, in theory, enhance extracellular ADO production and may in fact inappropriately propagate treatment resistance. This may account for the observation that a portion of MM patients do not respond to antibody therapy and nearly all patients will ultimately become refractory to treatment.^70,71^ Targeting CD38 enzymatic activity in the TME presents a potential new strategy for combating the immunosuppressive effects that contribute to treatment resistance to antibody therapy in high-risk NB. We postulate that this approach may also be of value in other cancers featuring CD38 expression and disproportionate NK cell populations.

The known CD38 inhibitor 14 increased NAD^+^ levels *in vitro* and *in vivo*, but was developed for use in metabolic disease, and was not evaluated in the framework of cancer immunotherapy.^19,20,48,49^ We have explored a quinazoline dihydropyrimidine scaffold from a hit molecule, compound 1, previously identified by our laboratory as an inhibitor of CD38-hydolase activity.^20^ Preliminary SAR analysis of 12 commercially available or newly synthesized molecules, indicates that the dihydropyrimidine moiety (1–3) or its isosteric equivalent (4, 5 and 7) is optimal for activity. Alkyl substitution at the 6- and 8-positions of the quinazoline ring are well tolerated. Furthermore, substitution at the 4- and 6-positions of the dihydropyrimidine ring is also tolerated without severely diminishing activity. This appears to be supported by *in silico* modeling (Fig. 5), which suggests that the 6-position on the quinazoline ring and the 6-position on the dihydropyrimidine ring are solvent exposed.

In using 14 as a positive control, we were surprised that the immunostimulatory effects of this known CD38 inhibitor (IC_50_ 78 nM) were significantly less than those of 2 (IC_50_ 1.9 μM) despite having a much lower IC_50_ value against CD38. One potential explanation for this observation may lie in the ultimate cellular location of the analogues. CD38 is known to function both as an intracellular and extracellular enzyme.^72^ We hypothesize that in addition to IC_50_, the activity of our compounds is dependent on the compartmentalization of 2*versus*14. It is possible that 14 penetrates into cells, where inhibition of CD38 would only marginally affect ADO levels in the tumor microenvironment. By contrast, 2 may not penetrate into cells as readily as 14, and thus its CD38 inhibitory activity would be extracellular. The observed effects of 2 on IFNγ levels and the associated immunostimulation likely result from a reduction of extracellular ADO levels. When an optimized analogue of 2 becomes available, we will undertake experiments to test this hypothesis.

Among the analogues structurally related to the parent molecule 1, the synthetic analog 2 exhibits the most potent activity against CD38 (IC_50_ 1.9 μM). Substitution of a methoxy substituent for the methyl group at the 8-position of the quinazoline ring improved potency more than 2-fold. *In silico* modeling (Fig. 5) suggests that there is a hydrogen bond interaction between the methoxy group of 2 and the aromatic adenosine moiety on NAD^+^ that may account for the increase in activity. We previously reported^20^ that compound 1 inhibits CD38 hydrolase activity *via* mixed inhibition. This observation is in agreement with data for the known CD38 inhibitor 14, which also exhibits mixed inhibition kinetics.^49^ For our *in silico* model in Fig. 5, we chose to use the PDB X-ray structure 2I66 (CD38 mutated at E226 in complex with NAD^+^) rather than PDB 4XJT, where CD38 is mutated at E226 and ADPR is covalently bound to the active site.^73^ The docking pose of 2 in PDB 2I66 strongly supports a mixed mechanism of inhibition, since 2 binds partially in the NAD^+^ catalytic pocket and partly outside of it. However, co-crystallization of 2 with CD38 complexed with NAD^+^ would be necessary to confirm this suggested binding pose.

Evaluation of compound 2 in PB NK cells suggest that small molecule inhibition of CD38 hydrolase activity promotes proliferation and prolonged viability of NK cells and increases extracellular secretion of IFNγ. The enhanced proliferation of NK cells was statistically significant after a 48 hour treatment (Fig. 6), and the observed increase in NK cell proliferation and increase in IFNγ secretion persisted throughout a 20 days treatment (Fig. 8). A previous report that CD38 knockout NK cells secreted more IFNγ relative to wild type NK cells^74^ coupled with our data suggest that CD38 enzymatic activity may be a source of NK cell exhaustion.^74^ Inhibition of CD38 hydrolase activity in NK cells might be a useful strategy for invoking an activated NK cell phenotype. Treatment of peripheral blood NK cells with 50 ng mL^−1^ ch14.18-IL2 and compound 2 exhibited enhanced cytotoxic effects of NK cells toward NB cells. While the mechanism of the observed cytotoxic effect has not yet been elucidated, these preliminary studies indicate that inhibition of CD38 hydrolase activity might be effective in enhancing NB immunotherapy used in clinic. Mechanistic studies, as well as the synthesis and evaluation of additional compounds related to 2 are ongoing concerns in our laboratory.

## Conclusions

In this report we describe the discovery, synthesis and biological characterization of a novel series of small molecule CD38 inhibitors for use in the treatment of NB. To our knowledge, these are the first small molecules designed for the stimulation of immune cells in cancer immunotherapy. Our studies have shown that the CD38 inhibitor 2 (IC_50_ 1.9 μM) is a potent inducer of IFNγ *in vitro* and that it promotes the proliferation of NK cells. Further, we have developed an *in vitro* NK/NB co-culture assay and demonstrated that 2 promotes a 14% decrease in the number of SHSY5Y NB cells after only 90 minutes. Importantly, although both 2 and the known CD38 inhibitor 14 produced significant increases in NAD^+^ levels,^20^ compound 2 was superior to 14 in terms of effects on IFNγ, NK cell proliferation and production of NK cell-induced cytotoxicity in SHSY5Y NB *in vitro*. Because the observed increase in NB cytotoxicity was mediated by inhibition of CD38 and the resulting increase in NK cell proliferation, this approach may be useful in other cancers that express CD38. In addition, agents related to 2 may be of use in preventing or delaying the development of resistance to ADCC observed with currently used mAb immune checkpoint inhibitors.

## Experimental

All reagents and dry solvents were purchased from Aldrich Chemical Co. (Milwaukee, WI), Sigma Chemical Co. (St. Louis, MO), VWR (Radnor, PA) or Fisher Scientific (Chicago, IL) and were used without further purification except as noted below. Triethylamine was distilled from potassium hydroxide and stored in a nitrogen atmosphere. Dry methanol, ethyl acetate, tetrahydrofuran, dimethyl formamide and hexane were either purchased (VWR) or prepared using a Glass Contour Solvent Purification System (Pure Process Technology, LLC, Nashua, NH). Microwave synthetic procedures were conducted on an Initiator 8 microwave synthesizer (Biotage, Charlotte, NC). Preparative scale chromatographic procedures were carried out using a Biotage Selekt chromatography system (Biotage, Charlotte, NC) fitted with silica gel 60 cartridges (230–440 mesh). Thin layer chromatography was conducted on Merck precoated silica gel 60 F-254. Compound 14 was purchased from Selleckchem (Houston, TX), and compounds 1, 4–9, and 11–13 were purchased from Vitas-M Laboratory (Champaign, IL). Compound 10 was obtained from the SC^3^ collection from the MUSC drug discovery core. All ^1^H and ^13^C-NMR spectra were recorded on a Bruker Avance 600 MHz spectrometer, and all chemical shifts are reported as d values referenced to TMS or DSS. Splitting patterns are indicated as follows: s, singlet; d, doublet; t, triplet; m, multiplet; br, broad peak. In all cases, ^1^H-NMR, ^13^C-NMR and MS spectra were consistent with assigned structures, and ^13^C peak assignments appear on the spectrum. Mass spectra were recorded by LC/MS on a waters UPLC/MS system with a model QDa mass spectrometer detector. Prior to biological testing procedures, all compounds were determined to be >95% pure by UPLC chromatography (9 : 1 H_2_O: acetonitrile, +0.1% formic acid to 1 : 9 H_2_O/acetonitrile +0.1% formic acid over 8 minutes) using a waters acquity H-series ultrahigh-performance liquid chromatograph fitted with a C18 reverse-phase column (Acquity UPLC BEH C18 1.7 M, 2.1 × 100 mm).

### Experimental procedures and compound characterization

#### 8-Methoxy-2,2,4,6-tetramethyl-1,2-dihydroquinoline (16b)

Scandium triflate (0.34 g, 0.79 mmol) was added to a flame dried, 3-neck 250 mL rbf equipped with stir bar and reflux condenser and stirred in anhydrous acetone (35.0 mL) and anhydrous acetonitrile (45.0 mL) for 15 minutes at room temperature. 2-Methoxy-4-methylaniline (15b) (5.00 g, 36.45 mmol) was added to the stirring solution and the reaction was heated to 88 °C for 72 hours. The reaction was concentrated to an oil and purified by flash column chromatography using hexanes/EtOAc to afford 8-methoxy-2,2,4,6-tetramethyl-1,2-dihydroquinoline (16b) as a pale-yellow oil (2.95 g, 37.1%). *R*_f_ = 0.57 (9 : 1 Hex : EtOAc). ^1^H NMR (DMSO-D6, 600 MHz): *δ* 1.16 (s, 6H), 1.86 (s, 3H), 2.15 (s, 3H), 3.72 (s, 3H), 4.63 (s, 1H), 5.25 (d, 1H, *J* = 1.2), 6.46 (s, 1H), 6.53 (d, 1H, *J* = 1.2); ^13^C NMR (DMSO-D6, 150 MHz): *δ* 18.9, 21.3, 28.1, 31.0, 51.3, 55.8, 111.5, 116.6, 120.8, 123.8, 128.1, 129.0, 131.1, 145.5; UPLC retention time 6.5 min; ESI-MS for C_14_H_19_NO: [M + H]^+^ calculated 217.15, found 218.05.

#### 2,2,4,8-Tetramethyl-1,2-dihydroquinoline (16c)

Scandium triflate (0.63 g, 1.47 mmol) was added to a flame dried, 3-neck 250 mL rbf equipped with stir bar and reflux condenser and stirred in anhydrous acetone (26.0 mL) and anhydrous acetonitrile (45.0 mL) for 15 minutes at room temperature. *o*-Toluidine (15c) (5.00 g, 46.66 mmol) was added to the stirring solution and the reaction was heated to 88 °C for 48 hours. The reaction was concentrated to an oil and purified by flash column chromatography using hexanes/EtOAc to afford 2,2,4,8-tetramethyl-1,2-dihydroquinoline (16a) as a pale-yellow oil (0.68 g, 7.8%). *R*_f_ = 0.47 (9 : 1 Hex : EtOAc). ^1^H NMR (DMSO-D6, 600 MHz): *δ* 1.21 (s, 6H), 1.86 (s, 3H), 2.02 (s, 3H), 4.81 (s, 1H), 5.27 (d, 1H), 6.37 (t, 1H, *J* = 7.2 Hz), 6.78 (d, 1H, *J* = 7.8 Hz), 6.81 (d, 1H, *J* = 7.8 Hz); ^13^C NMR (DMSO-D6, 150 MHz): *δ* 17.7, 19.0, 31.4, 115.4, 119.7, 120.2, 121.6, 128.0, 128.7, 130.1, 142.2; UPLC retention time 6.8 min; ESI-MS for C_13_H_17_N: [M + H]^+^ calculated 187.14, found 188.24.

#### 8-Methoxy-1-(4,8-dimethylquinazolin-2-yl)guanidine (18b)

8-Methoxy-2,2,4,6-tetramethyl-1,2-dihydroquinoline (16b) (0.98 g, 4.51 mmol), 2-cyanoguanidine (0.49 g, 5.82 mmol) and 2 M HCl (2.2 mL) were added to a 2–5 mL microwave vial equipped with stir bar, capped with a pressurized cap, and heated at 105 °C for 40 min. The reaction was cooled to room temperature, allowing the formation of a white precipitate. The reaction was filtered, rinsing sparingly with 1 M HCl. The filtrand was sonicated in 10 mL (20% MeOH/20% NH_3_/DI water) for 15 minutes at room temperature. The solid was filtered and rinsed sparingly with MeOH to yield 1-(4,8-dimethylquinazolin-2-yl)guanidine (18b) as a white solid (0.87 g, 78.5%). *R*_f_ = Baseline (9 : 1 DCM : MeOH) ^1^H NMR (DMSO-D6, 600 MHz): *δ* 2.47 (s, 3H), 2.80 (s, 3H), 3.94 (s, 3H), 7.23 (s, 1H), 7.47 (s, 1H); ^13^C NMR (DMSO-D6, 150 MHz): *δ* 21.6, 21.8, 55.9, 114.8, 115.7, 120.7, 134.7, 139.0, 152.6, 157.2, 169.6; UPLC retention time 3.4 min; ESI-MS for C_12_H_15_N_5_O: [M + H]^+^ calculated 245.13, found 246.06.

#### 1-(4,8-Dimethylquinazolin-2-yl)guanidine (18c)

2,2,4,8-Tetramethyl-1,2-dihydroquinoline (16c) (0.586 g, 3.13 mmol), 2-cyanoguanidine (0.348 g, 4.14 mmol) and 2 M HCl (1.3 mL) were added to a 2–5 mL microwave vial equipped with stir bar, capped with a pressurized cap, and heated at 105 °C for 40 min. The reaction was cooled to room temperature, allowing the formation of a white precipitate. The reaction was filtered, rinsing sparingly with 1 M HCl. The filtrand was sonicated in 5 mL (20% MeOH/20% NH_3_/DI water) for 15 minutes at room temperature. The solid was filtered and rinsed sparingly with MeOH to yield 1-(4,8-dimethylquinazolin-2-yl)guanidine (18c) as a white solid (0.475 g, 70%). *R*_f_ = Baseline (9 : 1 DCM : MeOH) ^1^H NMR (DMSO-D6, 600 MHz): *δ* 2.53 (s, 3H), 2.81 (s, 3H), 7.36 (t, 1H, *J* = 7.8 Hz), 7.71 (d, 1H, *J* = 6.6 Hz), 7.96 (d, 1H, *J* = 7.8 Hz); ^13^C NMR (DMSO-D6, 150 MHz): *δ* 17.2, 21.6, 123.6, 124.7, 134.4, 147.8, 156.8, 171.1; UPLC retention time 3.1 min; ESI-MS for C_11_H_13_N_5_: [M + H]^+^ calculated 215.13, found 216.15.

#### 8-Methoxy-4,6-dimethyl-*N*-(4,4,6-trimethyl-1,4-dihydropyrimidin-2-yl)quinazolin-2-amine (2)

1-(4,8-Dimethylquinazolin-2-yl)guanidine (18b) (0.50 g, 2.04 mmol), 4-methylpent-3-en-2-one (350 μL, 2.71 mmol) and anhydrous dimethyl sulfoxide (2.0 mL) were added to a 2–5 mL microwave vial equipped with stir bar, flushed with nitrogen gas, capped with a pressurized cap, and heated at 100 °C for 24 hours. The reaction was poured over ice cold DI water (500 mL) and extracted with DCM (500 mL). The organic layer was separated, dried over MgSO_4_, filtered, and concentrated to a dark brown oil. Acetone was added dropwise until a fine yellow precipitate formed. The acetone was decanted and the crystals washed with acetone to yield 8-methoxy-4,6-dimethyl-*N*-(4,4,6-trimethyl-1,4-dihydropyrimidin-2-yl)quinazolin-2-amine (2) as a pale peach colored solid (0.016 g, 2.4%). *R*_f_ = 0.3 (9 : 1 DCM : MeOH). ^1^H NMR (DMSO-D6, 600 MHz): *δ* 1.32 (s, 6H), 1.78 (s, 3H), 2.43 (s, 3H), 2.66 (s, 3H), 3.93 (s, 3H), 4.50 (d, 1H, *J* = 1.2 Hz), 7.07 (d, 1H, *J* = 1.2 Hz), 7.31 (s, 1H); ^13^C NMR (DMSO-D6, 150 MHz): *δ* 18.0, 21.6, 22.0, 32.0, 51.0, 55.8, 103.9, 113.6, 115.5, 119.2, 129.9, 132.1, 139.1, 152.1, 152.9, 161.2, 167.8; UPLC retention time 4.7 min; ESI-MS for C_18_H_23_N_5_O: [M + H]^+^ calculated 325.19, found 326.29.

#### *N*-(4,8-Dimethylnaphthalen-2-yl)-4,4,6-trimethyl-1,4-dihydropyrimidin-2-amine (3)

1-(4,8-Dimethylquinazolin-2-yl)guanidine (18c) (0.457 g, 2.12 mmol), 4-methylpent-3-en-2-one (323 μL, 2.82 mmol) and anhydrous dimethyl sulfoxide (1.8 mL) were added to a 2–5 mL microwave vial equipped with stir bar, flushed with nitrogen gas, capped with a pressurized cap, and heated at 100 °C for 24 hours. The reaction was poured over ice cold DI water (300 mL) and extracted with DCM (300 mL). The organic layer was separated, dried over MgSO_4_, filtered, and concentrated to a dark brown oil. Acetone was added dropwise until a fine yellow precipitate formed. The acetone was decanted and the crystals washed with acetone to yield *N*-(4,8-dimethylnaphthalen-2-yl)-4,4,6-trimethyl-1,4-dihydropyrimidin-2-amine (3) as a pale yellow solid (0.110 g, 17.5%). *R*_f_ = 0.4 (9 : 1 DCM : MeOH). ^1^H NMR (DMSO-D6, 600 MHz): *δ* 1.34 (s, 6H), 1.79 (s, 3H), 2.52 (s, 3H), 2.72 (s, 3H), 4.53 (d, 1H, *J* = 0.6 Hz), 7.22 (t, 1H, *J* = 7.8 Hz), 7.61 (d, 1H, *J* = 7.2), 7.84 (d, 1H, *J* = 7.8), 10.13 (s, 1H); ^13^C NMR (DMSO-D6, 150 MHz): *δ* 17.5, 18.0, 21.8, 32.0, 104.0, 118.7, 122.4, 123.1, 129.9, 132.2, 133.1, 148.1, 152.7, 161.6, 169.0. ESI-MS for C_17_H_21_N_5_: [M + H]^+^ calculated 295.18, found 296.18.

#### Human recombinant CD38 enzyme assay

Compounds were screened for inhibition against CD38 hydrolase activity in a human recombinant CD38 fluorometric assay. Recombinant CD38 was diluted in assay diluent (PBS, 0.02% Tween-20, pH 7.4) to a 4× working concentration (40 nM). Test compounds were diluted in assay diluent to a working concentration of 200 μM. Working CD38 (25.0 μL) and the 4× test compound (25.0 μL) were pipetted into a black 96-well microplate and incubated for 15 minutes at 25 °C on an orbital shaker. Following incubation, 20 μM εNAD^+^ (50 μL) was pipetted into each well and fluorescence intensity was measured kinetically for 10 minutes (ex/em: 300/410 nm) using a Molecular Devices SpectraMax iD3 plate reader. Final concentrations of all components were: 10 nM recombinant CD38, 50 μM test compound (50 nM for control compound 3), and 10 μM εNAD^+^. Means for data points at 1 minute 49 seconds were normalized to vehicle treated control. Values were expressed as percent remaining activity of CD38. Each assay was conducted in technical and experimental triplicate and compared to literature reported values of 14.

Compounds were also tested for inhibition against CD38 cyclase activity at a concentration of 50 μM using the same enzyme assay, where a 2 × 50.0 μM working solution of NGD^+^ was used in place of εNAD^+^. No compounds were found to exhibit cyclase inhibitory activity greater than 20%.

#### Criteria for IC_50_ determination

Compounds were evaluated for structural similarity to compound 1 and those with Tanimoto coefficients <0.8 were subjected to preliminary testing at a concentration of 50.0 μM.^20^ Any test compounds that exhibited <65.0% remaining CD38 hydrolase activity in preliminary testing were further evaluated for IC_50_'s in our recombinant enzyme assay as previously described.^20^ Compounds with Tanimoto coefficients >0.8 were not subjected to preliminary testing and were evaluated for IC_50_'s.

#### *In silico* docking and molecular dynamics

Modeling, simulations and structural visualizations were performed using MOE 2019 (Chemical Computing Group ULC, Montreal, CA) based on RCSB Protein Data Bank structure 2I66.^72^ The protein was protonated at *T* = 310 K, pH 7.0, salt at 200 mM using GB/VI electrostatics. Docking simulations used flexible receptor and flexed the ligand, while docking targeted the active site. For each docking simulation initial placement calculated 50 poses using triangle matching with London dG scoring, the top 5 poses were refined using forcefield Amber10:ETH and Affinity dG scoring (Escore2). The top pose was used then refined using molecular dynamics. Molecular dynamics used the NPA algorithm and the Amber10:ETH forcefield. Solvent was a water droplet with 0.1 M NaCl and used 9518 solvent molecules. Simulation protocol was an equilibrium step for 100 ps at 300 K and a production step for 500 ps at 300 K with a step time of 0.5 ps.

#### Cell assays

Human PB NK cells were cultured on 6-well cell culture plates in Immunocult-XF T cell expansion medium (StemCell Technologies, Vancouver, BC, Canada) supplemented with 1× penicillin/streptomycin (VWR, Radnor, PA), 500 IU mL^−1^ IL-2 (StemCell Technologies), 0.2 μL mL^−1^ CD2/CD3/CD28 T cell activator (StemCell Technologies), and 10 ng mL^−1^ IL-15 (StemCell Technologies). Culture medium was replaced every 3 days, and cells were kept at a concentration no less than 1 × 10^6^ cells mL^−1^. Human PB NK cells were expanded for 14–21 days prior to cell experiments. PB NK cells were purchased from StemCell Technologies. Cells were purified *via* FACS sorting using the surface marker CD56. All cell donors were ≥90% pure. SHSY5Y-GFP cells were cultured in RPMI 1640 (VWR, Radnor, PA) supplemented with 10% FBS (VWR, Radnor, PA) and 1× penicillin/streptomycin in T75 vented cell culture flasks. Cells were split every 2–3 days using trypsin (0.25%) (VWR, Radnor, PA).

#### Cell viability/proliferation assay

Human PB NK cells were plated (50 μL per well) at 40 000 cells per well in RPMI 1640 supplemented with 10% FBS, 1× penicillin/streptomycin, and 100 IU mL^−1^ IL-2 on a black, clear-bottom, 96-well microplate. Test compounds were dissolved in DMSO and diluted to a 2× working solution with cell culture medium. Working test compound or DMSO vehicle (50 μL) was added to cells and the cells were incubated at 37 °C, 5% CO_2_ for 48 hours. Following incubation, cells were treated with Hoechst (10 μL, final concentration 1 μg mL^−1^), and imaged using a BioTek Cytation 5 imager. Cells were kept at 37 °C, 5% CO_2_ during imaging. Each well was imaged in 4 quadrants at 4× magnification using the DAPI fluorescence channel. The fluorescence intensity was measured as the sum of integrated fluorescence, with treatment wells normalized to vehicle treated controls and data expressed as the percent difference of PB NK nuclear area. Each assay was tested in technical triplicate.

#### Quantitation of IFNγ

Human PB NK cells were plated at 49 000 cells per well (60 μL per well) on a white, clear-bottom, 96-well microplate in Immunocult-XF T cell expansion medium supplemented with 1× penicillin/streptomycin, 500 IU mL^−1^ IL-2, 10 ng mL^−1^ IL-15, 0.2 μL mL^−1^ CD2/CD3/CD28 T cell activator. Test compounds were dissolved in DMSO and diluted to a 4× working solution. Working test compound was added to the cells (20 μL) and the cells were incubated at 37 °C, 5% CO_2_ for 24 hours. The last 3 columns on the microplate were left empty for IFNγ standards. The provided human IFNγ standard (10 μg mL^−1^) was diluted to 10 000 pg mL^−1^ using cell culture medium. The 10 000 pg mL^−1^ solution was serially diluted 3.33-fold for a total of 7 standard concentrations. The IFNγ standards were plated (80 μL) in the empty columns (in triplicate) following incubation. A 5× solution of both antibodies was prepared by combining the Lumit Anti-hIFN-γ-mAb-LgBiT (24 μL) and the Lumit Anti-hIFN-γ-mAb-SmBiT (24 μL) with cell culture medium (2.4 mL). The 5× antibody solution (20 μL) was pipetted into each well. The microplate was briefly mixed for 15 seconds on a shaker at 250 rpm and incubated for 90 minutes at 37 °C, 5% CO_2_. Following incubation, the microplate was allowed to rest at 25 °C for 15 minutes. The Lumit detection substrate (160 μL) was diluted with Lumit detection buffer B (3040 μL) and pipetted into each well (25 μL). The microplate was allowed to incubate for 5 minutes at 25 °C and then the luminescence was measured using a Molecular Devices SpectraMax iD3 plate reader. A standard curve with averaged RLU measurements of the IFNγ concentrations was determined with GraphPad Prism using 4-parameter logistic curve fitting. The test sample IFNγ concentrations were interpolated using the standard curve. Data was presented as percent difference relative to vehicle treated control. Each assay was tested in technical triplicate.

#### Optimization of SHSY5Y-GFP cell culture conditions

SHSY5Y-GFP cells were plated at 20 000, 30 000, 40 000, and 50 000 cells per well with 6 test wells each. Cells were incubated for 24 hours and imaged with a BioTek Cytation 5 at 20× with 4 fields per well using brightfield and GFP channels. The data is presented as the sum of SHSY5Y-GFP cells in imaged fields as a function of cells per well (Fig. 9A). Optimal plating density was determined to be between 20 000 and 30 000 cells per well. Additionally, we wanted to rule out proliferative effects in SHSY5Y-GFP cells treated with 2. Cells were plated at 20 000 or 30 000 cells per well and treated with varying concentrations of 2 or vehicle control for 24 hours and imaged as described above. Data was then expressed as the sum of SHSY5Y-GFP cells in imaged fields as a function of the concentration of 2 (Fig. 9B). A cell number difference of <4% was observed for vehicle-treated cells and cells treated with 1.0 μM 2 at both 20 000 cells per well and 30 000 cells per well.

#### Optimization of ch14.18-IL2 levels for co-culture assay

To conserve PB NK cells, the concentration of ch14.18-IL2 was optimized to elicit a sufficient response using an effector cell : target cell ratio of 1 : 1. SHSY5Y-GFP cells were plated at 25 000 cells per well and PB NK cells stained with cell tracker deep red were added along with either 50 ng mL^−1^ or 125 ng mL^−1^ ch14.18-IL2. Cells were incubated for 90 minutes and the sum of GFP integrated fluorescence was measured using a BioTek Cytation 5 imager.

#### SHSY5Y-GFP and PB NK cell co-culture assay

SHSY5Y-GFP NB cells were plated at a concentration of 25 000 cells per well (100 μL) for 12 hours on a black, clear-bottom, 96-well microplate. PB NK cells were washed with PBS and resuspended in 5 μM cell tracker deep red in RPMI 1640 without FBS for 30 minutes at 37 °C, 5% CO_2_. Following incubation, cells were washed with PBS and resuspended in RPMI 1640 supplemented with 10% FBS. Compounds were dissolved in DMSO and diluted to a 4 μM working solution with cell culture medium. Ch14.18-IL2 was diluted in cell culture medium to a 200 ng mL^−1^ working solution. SHSY5Y-GFP cell medium was aspirated and PB NK cells (50 μL), compound (25 μL), and ch14.18-IL2 (25 μL) were added to each well. Cells were incubated at 37 °C, 5% CO_2_ for 90 minutes. Cells were imaged at 20×, 4 fields per well, using bright field, GFP, and Cy 5 channels using a BioTek Cytation 5 imager. The sum integration of GFP area was measured and normalized to vehicle-treated control. Each assay was tested in technical triplicate.

## Data availability

All compound characterization data including ^1^H and ^13^C NMR, mass spectra and UPLC traces are available in the ESI.†

## Author contributions

Catherine M. Mills: conceptualization, methodology, validation, formal analysis, investigation, data curation, writing original draft, visualization. Thomas Z. Benton: conceptualization, methodology, validation, formal analysis. Ivett Piña: investigation, data curation, formal analysis. Leticia Reyes: investigation, data curation, formal analysis. Nathan G. Dolloff: formal analysis, resources, data curation, formal analysis. Yuri K. Peterson: conceptualization, methodology, validation, formal analysis, investigation, data curation, visualization. Patrick M. Woster: conceptualization, methodology, validation, formal analysis, resources, data curation, writing review and editing, visualization, supervision, project administration, funding acquisition.

## Conflicts of interest

There are no conflicts to declare.

## Supplementary Material

SC-014-D2SC05749B-s001
