# Supplementary material for: Stimulation of natural killer cells with small molecule inhibitors of CD38 for the treatment of neuroblastoma
Source: Chem Sci. 2023 Jan 30;14(8):2168–82. doi: 10.1039/d2sc05749b (PMC9945084; doi:10.1039/d2sc05749b)
Supplement: SC-014-D2SC05749B-s001 [file SC-014-D2SC05749B-s001.pdf]

Electronic Supplementary Material (ESI) for Chemical Science.
This journal is © The Royal Society of Chemistry 2023

Stimulation of Natural Killer Cells with Small Molecule CD38 Inhibitors for the Treatment of Neuroblastoma

*Catherine M. Mills,[a] Thomas Z. Benton,[a] Ivett Piña,[a] Megan J. Francis,[a] Leticia Reyes,[b] Nathan G. Dolloff,[b] Yuri K. Peterson[a] and Patrick M. Woster[a]**

[a]Department of Drug Discovery and Biomedical Sciences, Medical University of South Carolina; 70 President St, Charleston, SC, 29425, USA

[b]Department of Cell and Molecular Pharmacology and Experimental Therapeutics, Medical University of South Carolina; 70 President St, Charleston, SC, 29425, USA

Supplementary Information

^{1}H-NMR and ^{13}C-NMR Data for Compounds 16b, 16c, 18b, 18c, 2 and 3

Compound **16b**

CMM-42a-i-in DMSO

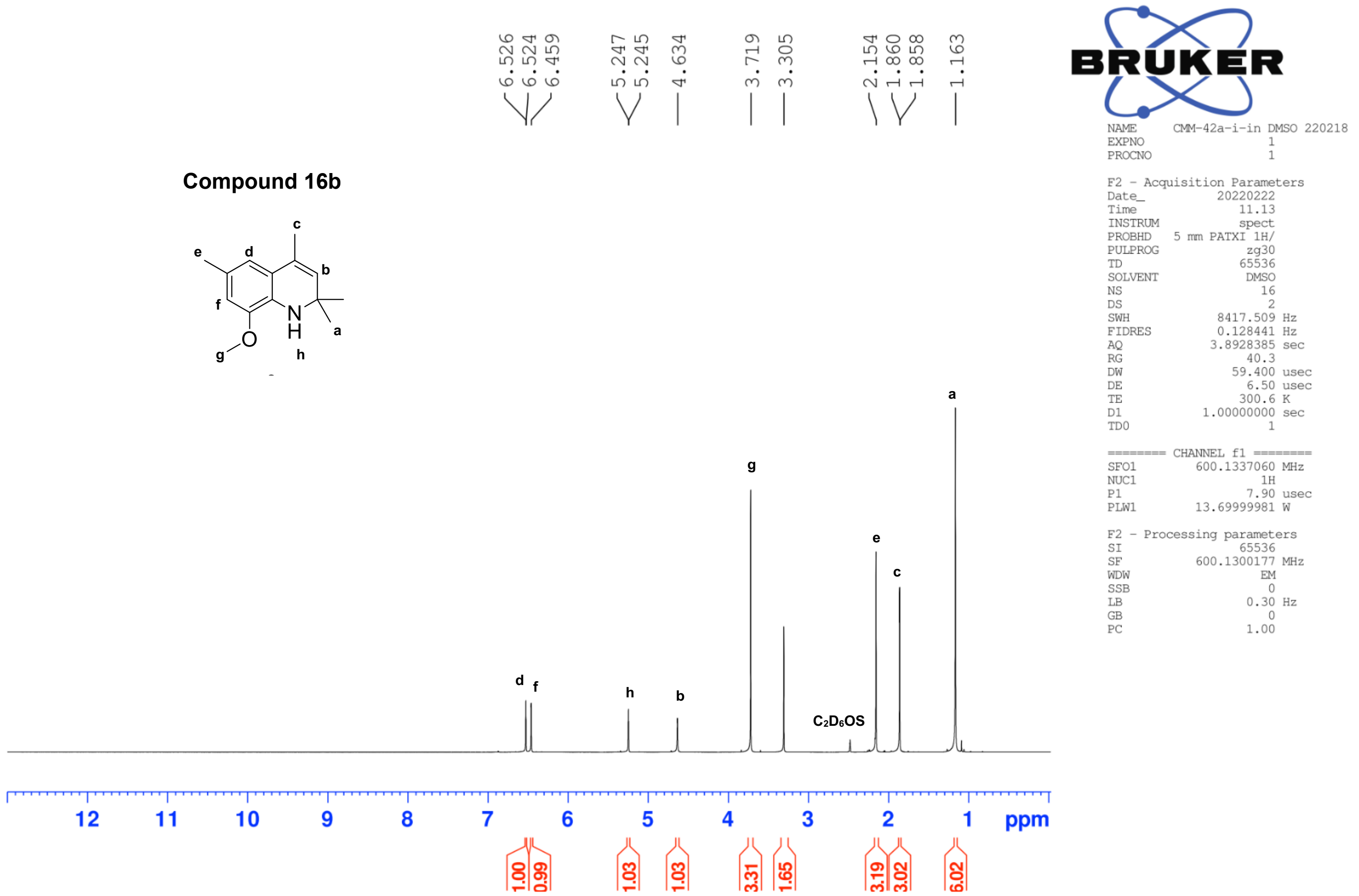

CMM-42a-i-in DMSO

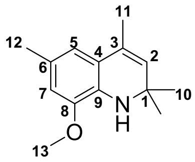

Compound 16b

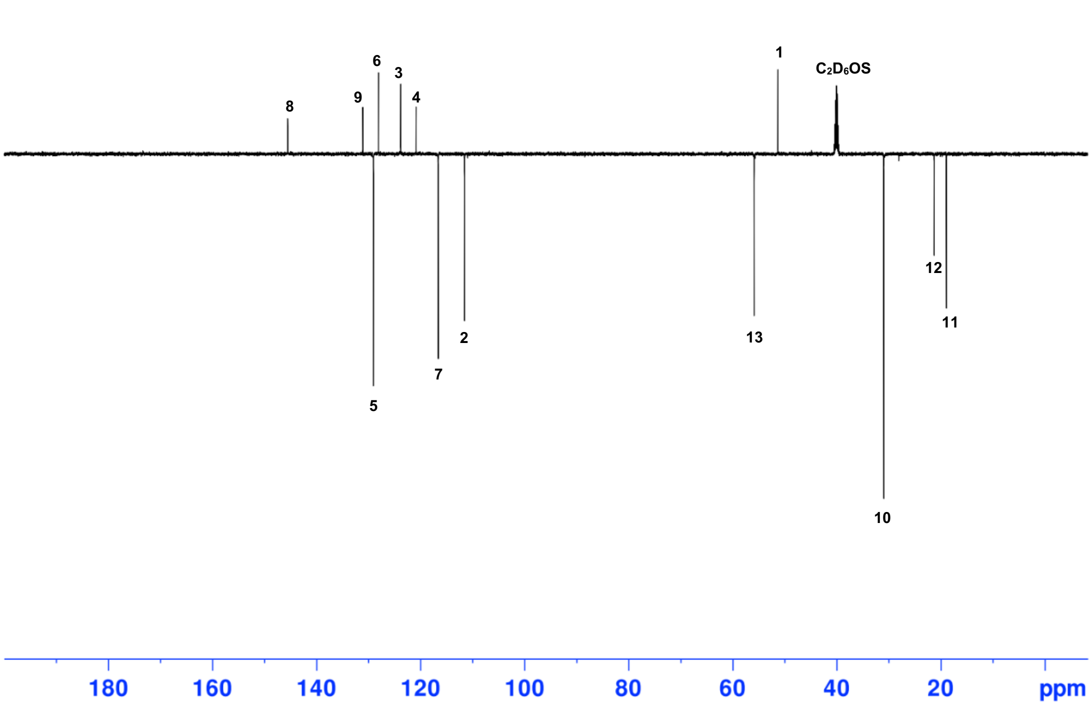


```
Current Data Parameters
NAME      CMM-42a-i-in DMSO 220218
EXPNO                    3
PROCNO                   1

F2 - Acquisition Parameters
Date_             20220222
Time                 11.58
INSTRUM              spect
PROBHD    5 mm PATXI 1H/
PULPROG               jmod
TD                   65536
SOLVENT               DMSO
NS                     881
DS                       4
SWH             36057.691 Hz
FIDRES           0.550197 Hz
AQ              0.9087659 sec
RG                    2050
DW                  13.867 usec
DE                    6.50 usec
TE                   301.5 K
CNST2          145.0000000
CNST11           1.0000000
D1              2.00000000 sec
D20             0.00689655 sec
TD0                      1

======== CHANNEL f1 ========
SFO1           150.9178988 MHz
NUC1                   13C
P1                   11.80 usec
P2                   23.60 usec
PLW1          202.10000610 W

======== CHANNEL f2 ========
SFO2           600.1324005 MHz
NUC2                    1H
CPDPRG[2           waltz16
PCPD2                70.00 usec
PLW2           13.69999981 W
PLW12           0.17449000 W

F2 - Processing parameters
SI                   32768
SF             150.9028090 MHz
WDW                     EM
SSB                      0
LB                    1.00 Hz
GB                       0
PC                    1.40
```

Compound 16b

LC-MS

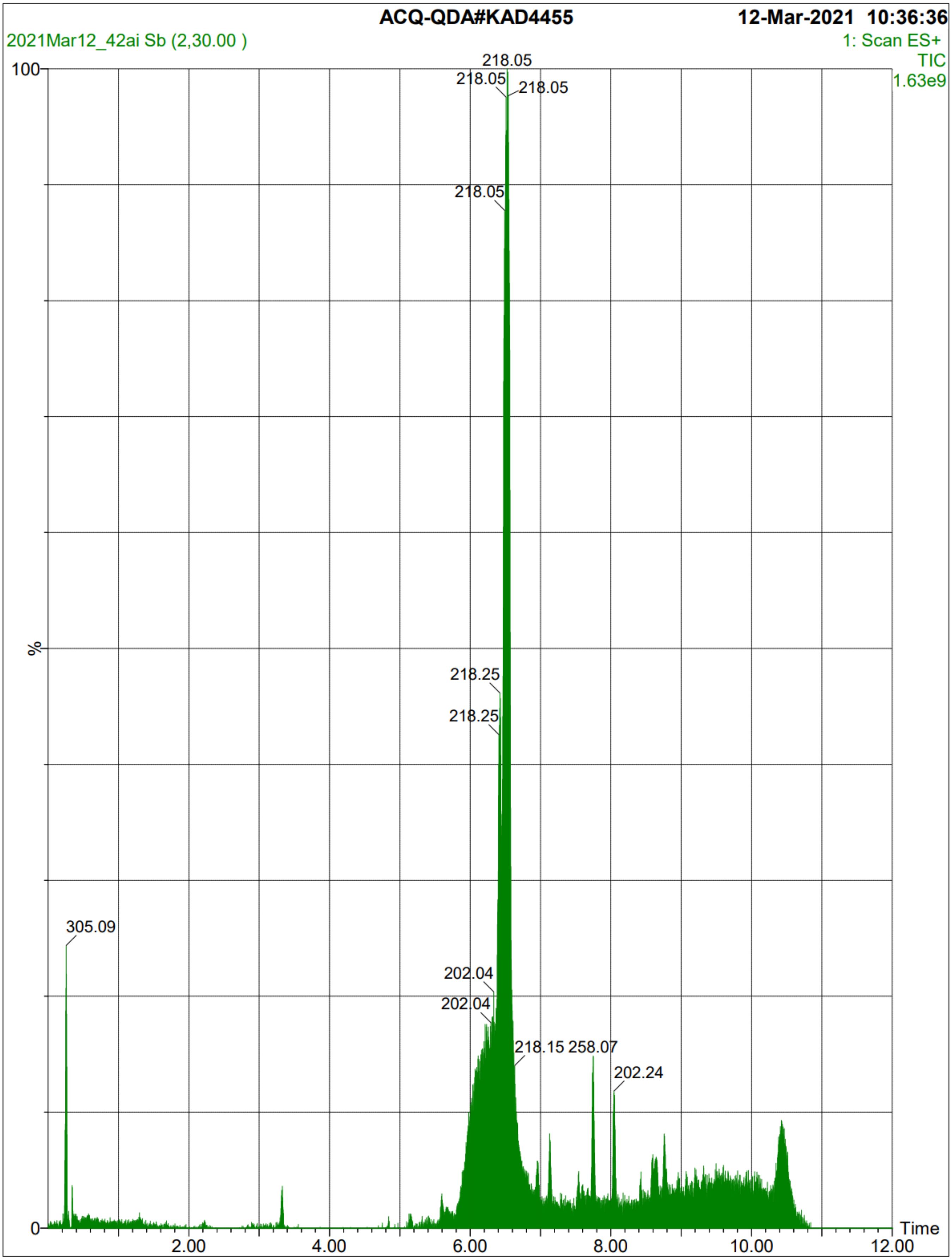

MS

Compound 16b

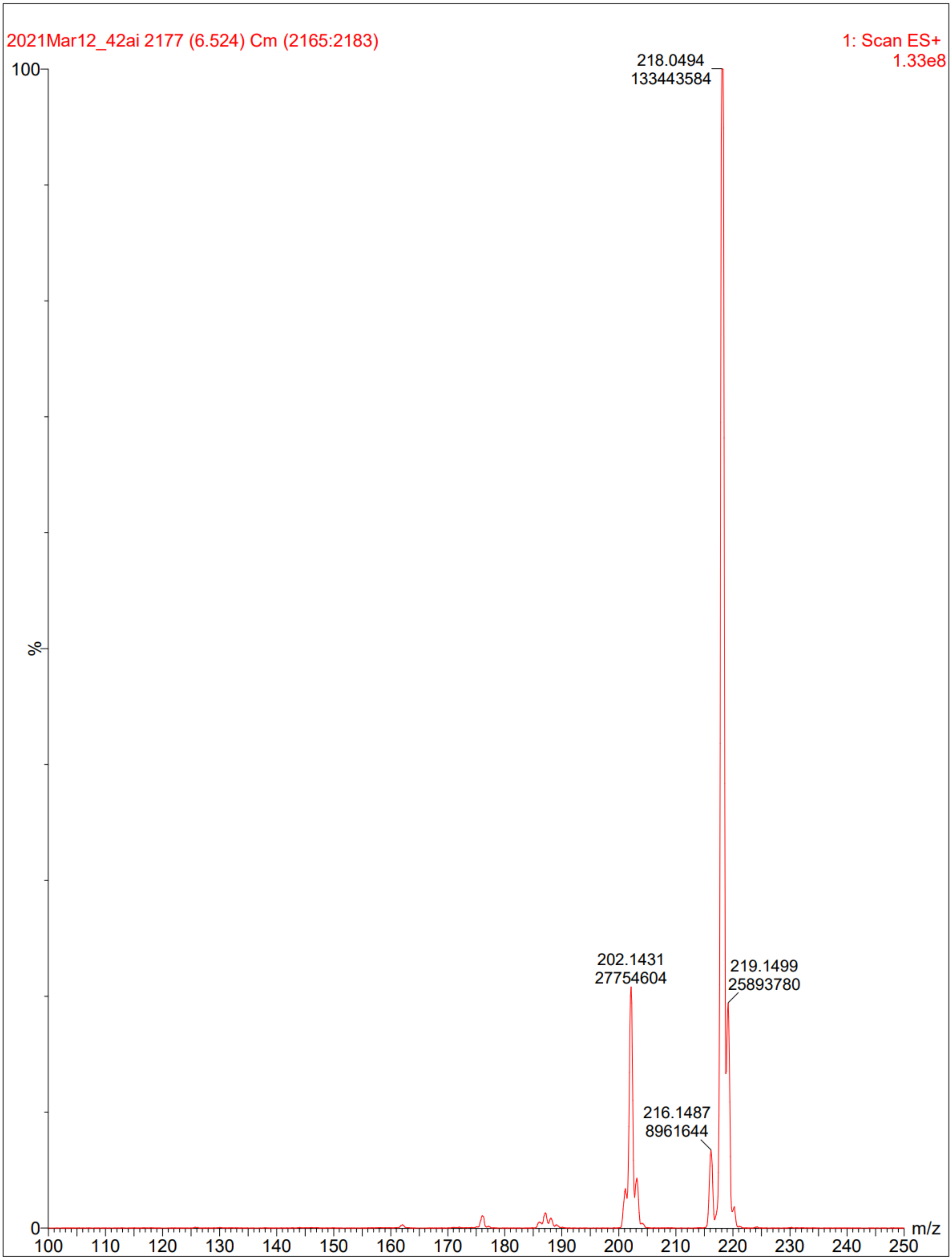

Compound 16b

Default Individual Report

SAMPLE INFORMATION

Sample Name:	42ai	Acquired By:	System
Sample Type:	Unknown	Sample Set Name	CMM210108_42 analogs
Vial:	1:A,6	Acq. Method Set:	Default
Injection #:	1	Processing Method	Default
Injection Volume:	5.00 ul	Channel Name:	PDA Max Plot 190.0 - 800.0
Run Time:	12.0 Minutes	Proc. Chnl. Descr.:	PDA MaxPlot (190.0 nm to 800.0
Date Acquired:	10/6/2022 8:35:24 AM EDT		
Date Processed:	10/6/2022 9:44:26 AM EDT		

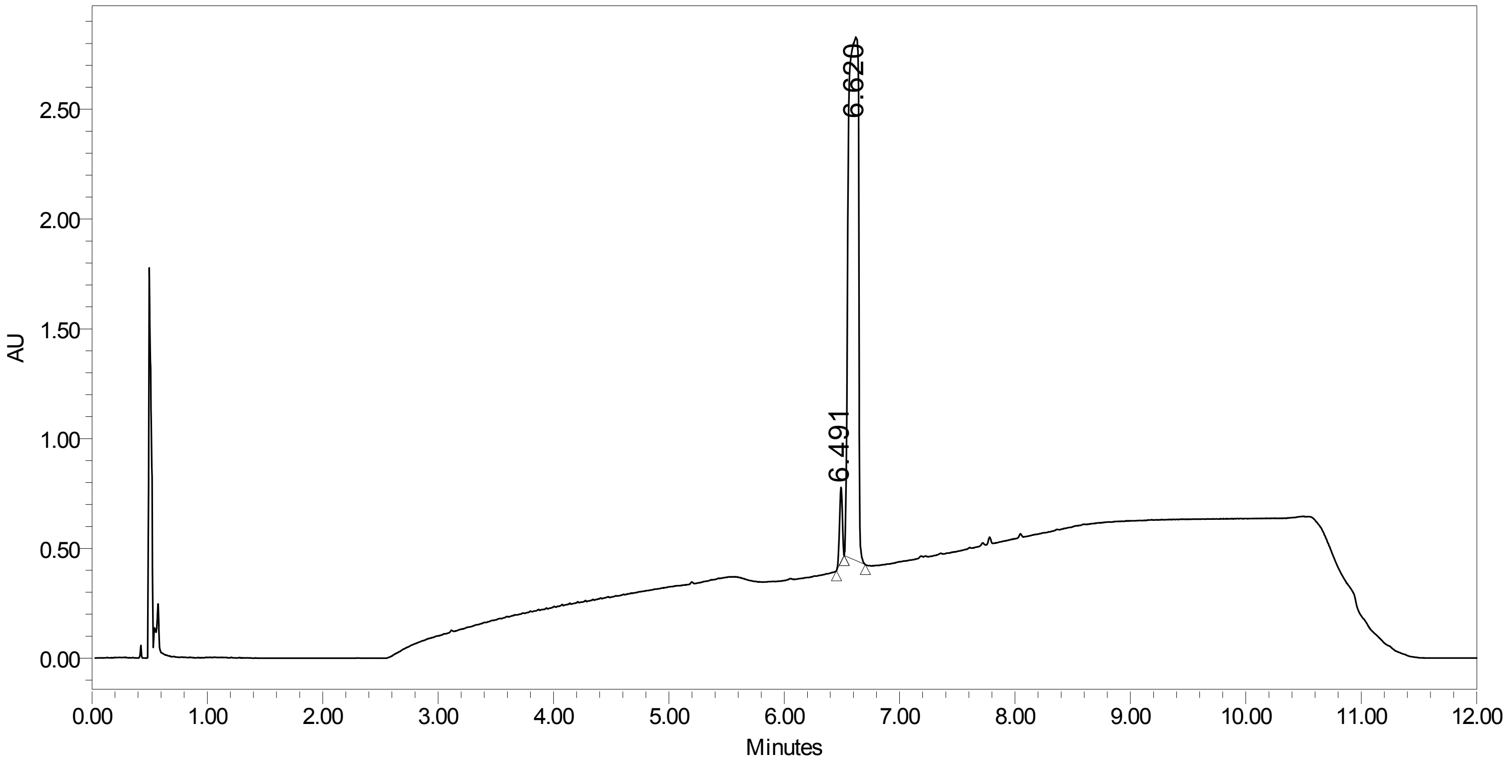

	RT	Area	% Area	Height
1	6.491	577324	3.92	338388
2	6.620	14135264	96.08	2384331

PDA Result Table

	Name	RT	Purity1 Angle	Purity1 Threshold	Match1 Spect. Name	Match1 Angle	Match1 Threshold
1		6.491	0.759	0.777			
2		6.620	6.725	4.005			

Compound **16c**

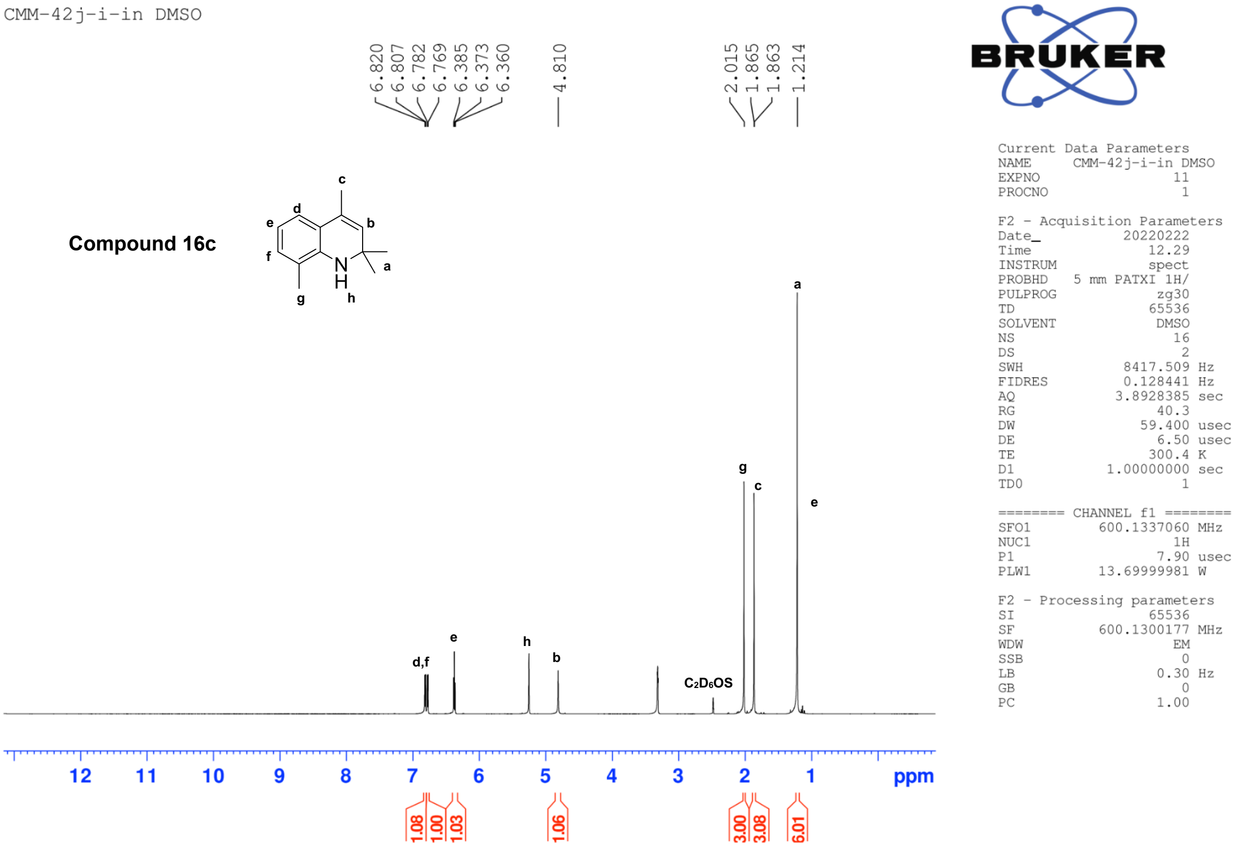

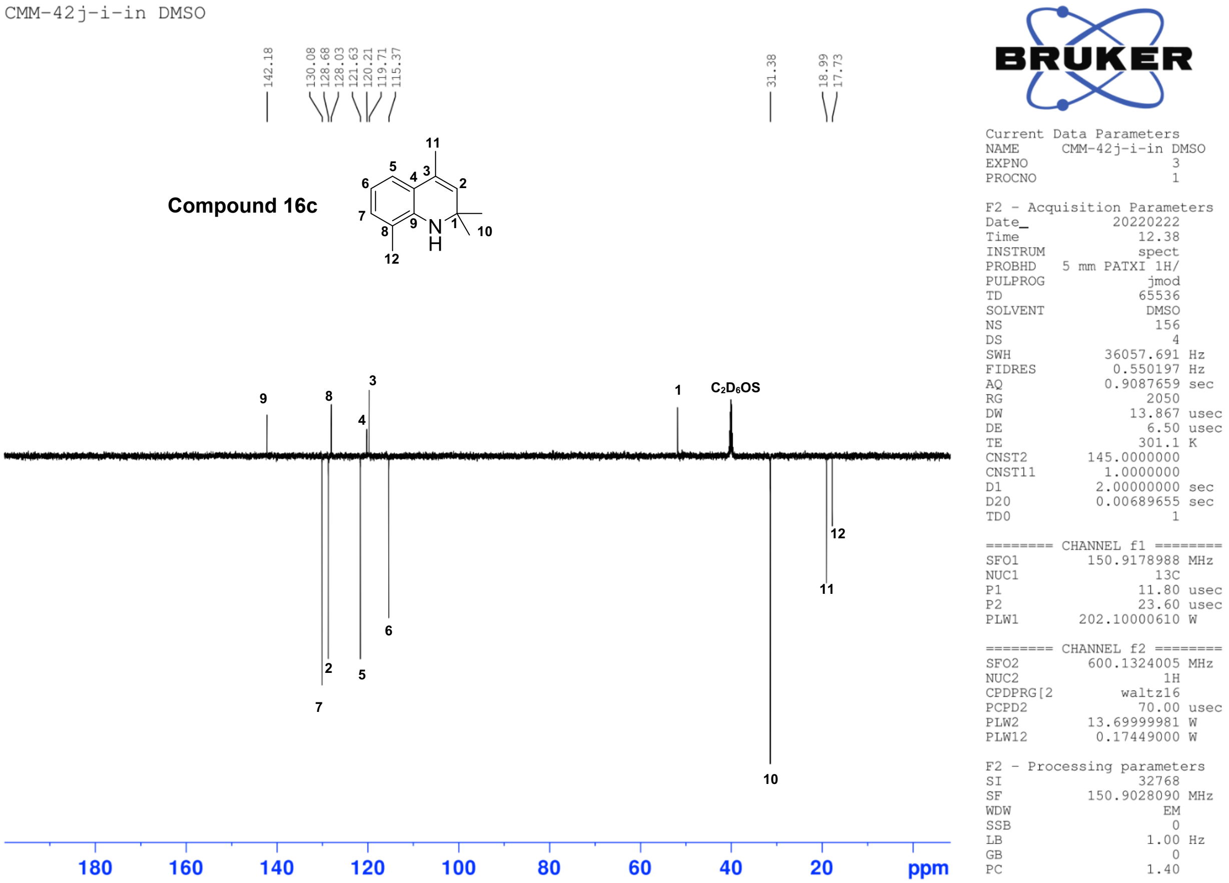

Compound 16c

LC-MS

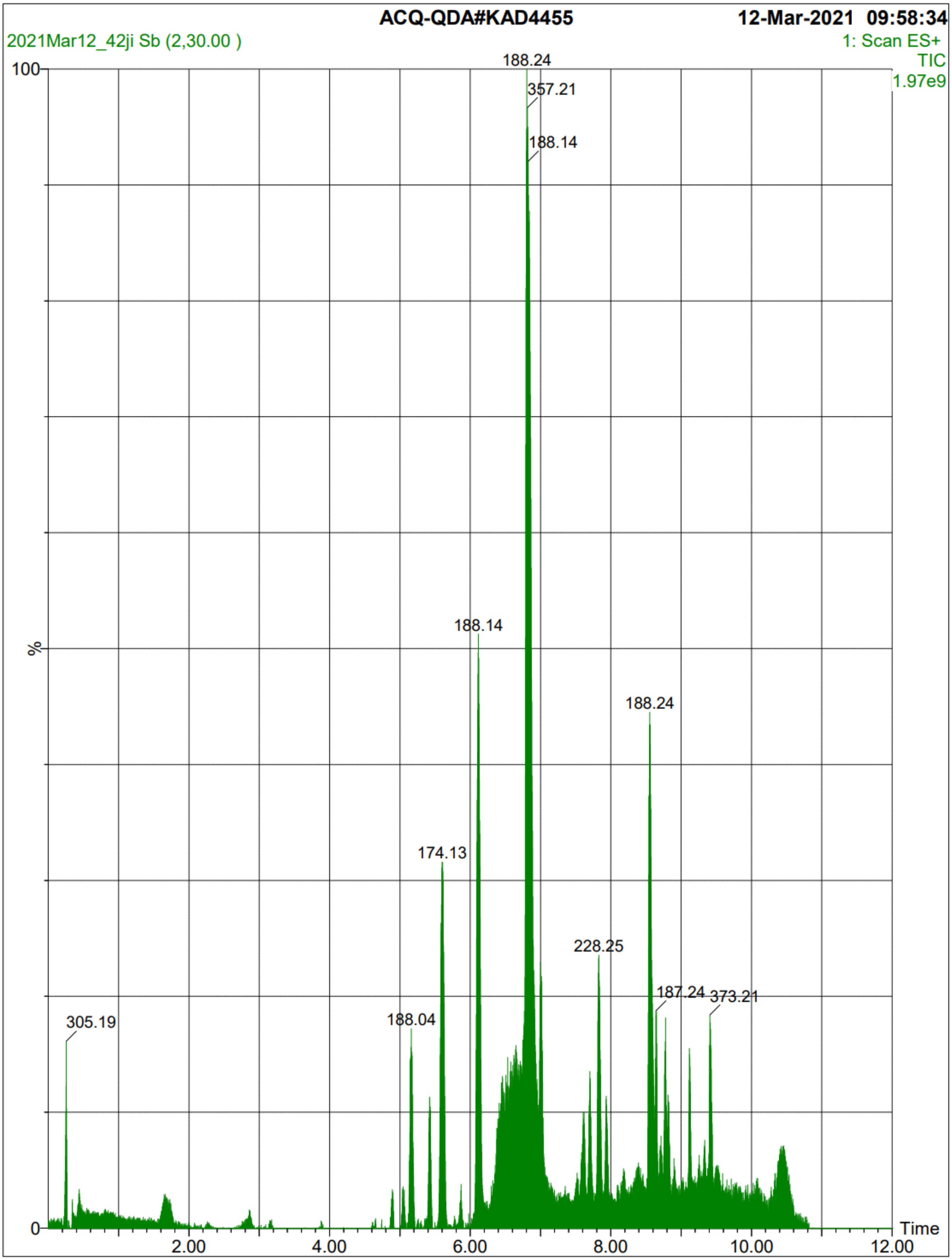

Compound 16 c

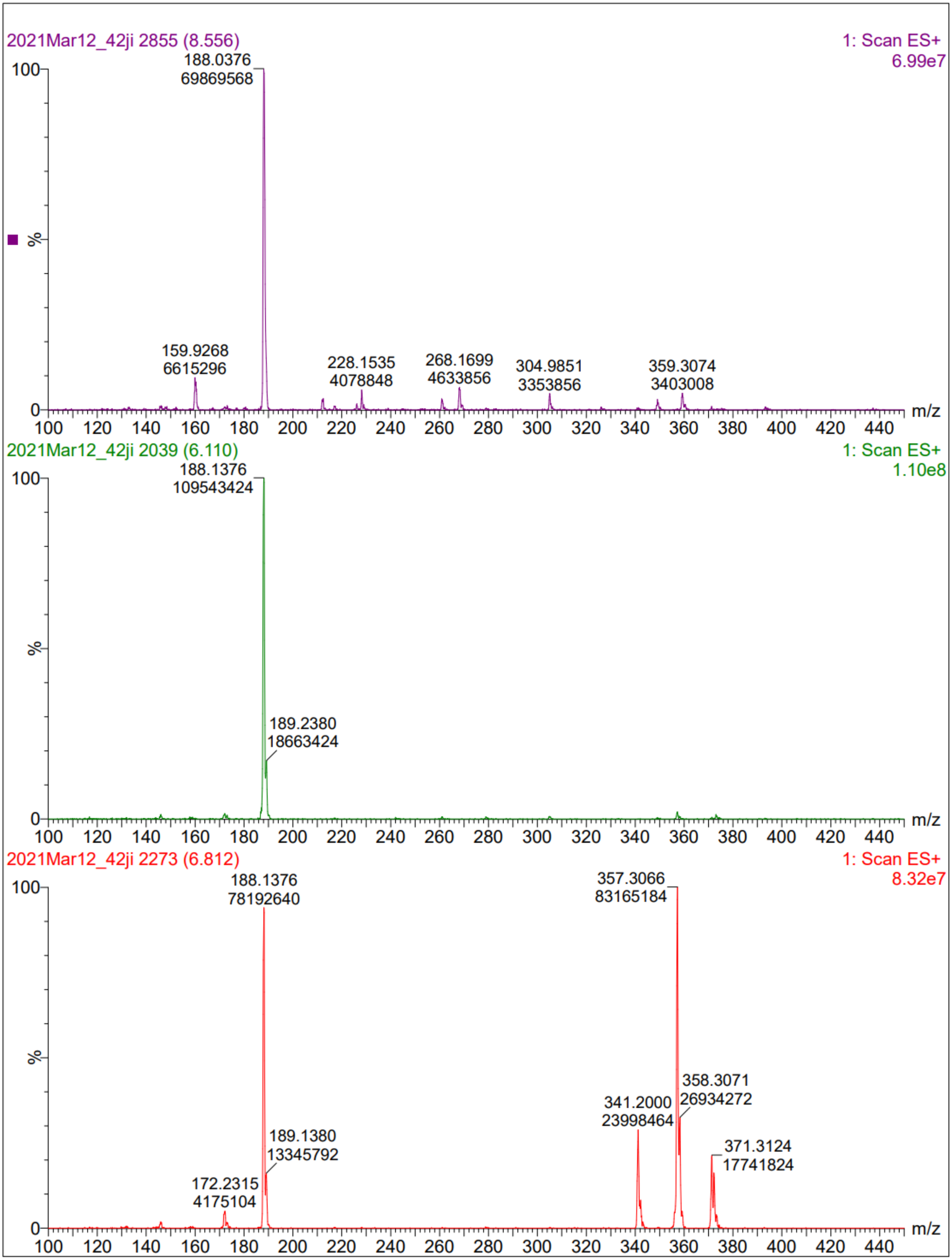

Compound 16c

Default Individual Report

SAMPLE INFORMATION

Sample Name:	42j_i	Acquired By:	System
Sample Type:	Unknown	Sample Set Name	CMM221005_42 analogs
Vial:	1:A,3	Acq. Method Set:	Default
Injection #:	1	Processing Method	Default
Injection Volume:	5.00 ul	Channel Name:	PDA Max Plot 190.0 - 800.0
Run Time:	12.0 Minutes	Proc. Chnl. Descr.:	PDA MaxPlot (190.0 nm to 800.0
Date Acquired:	10/5/2022 2:08:31 PM EDT		
Date Processed:	10/5/2022 2:22:05 PM EDT		

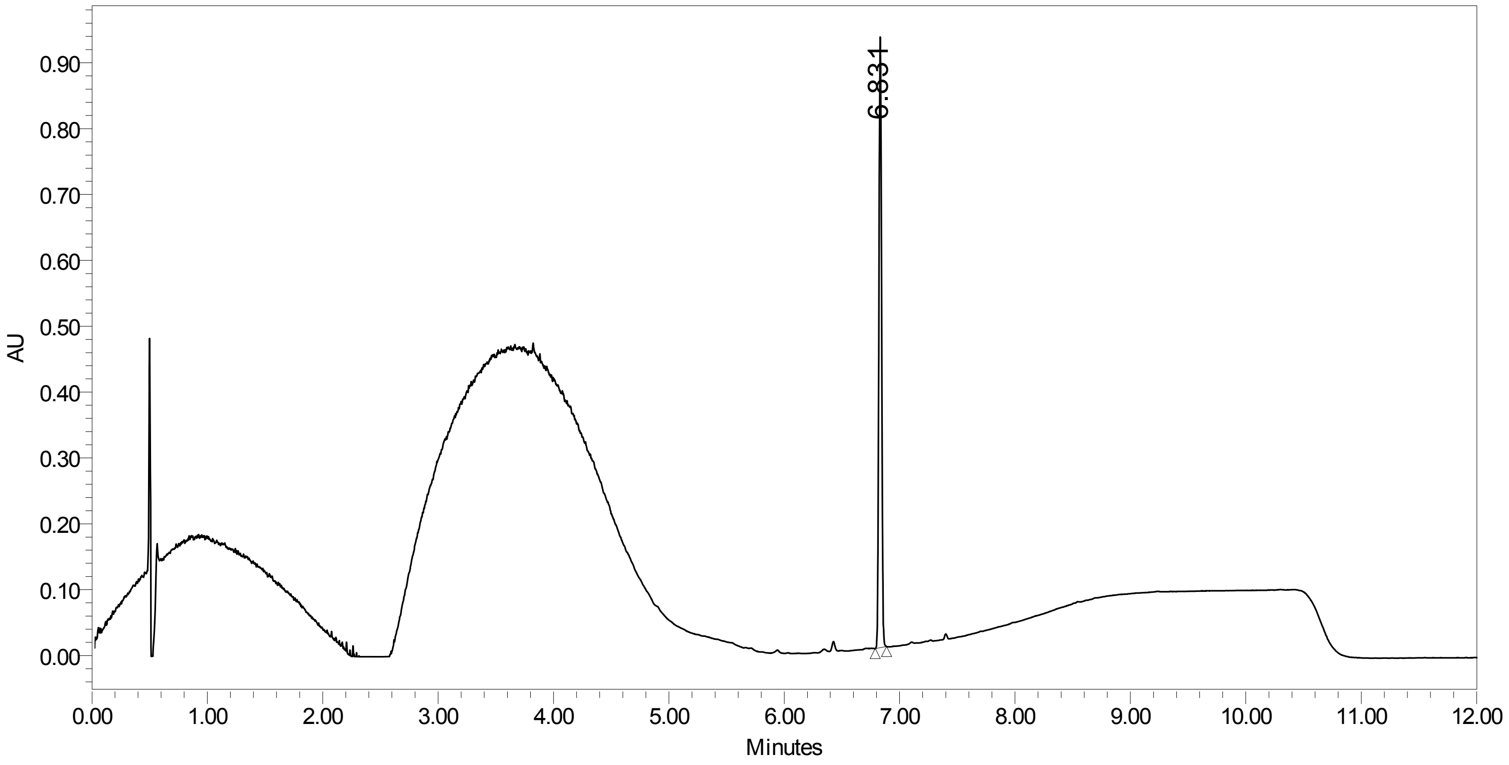

	RT	Area	% Area	Height
1	6.831	1501833	100.00	927736

PDA Result Table

	Name	RT	Purity1 Angle	Purity1 Threshold	Match1 Spect. Name	Match1 Angle	Match1 Threshold
1		6.831	1.435	0.466			

Compound **18b**

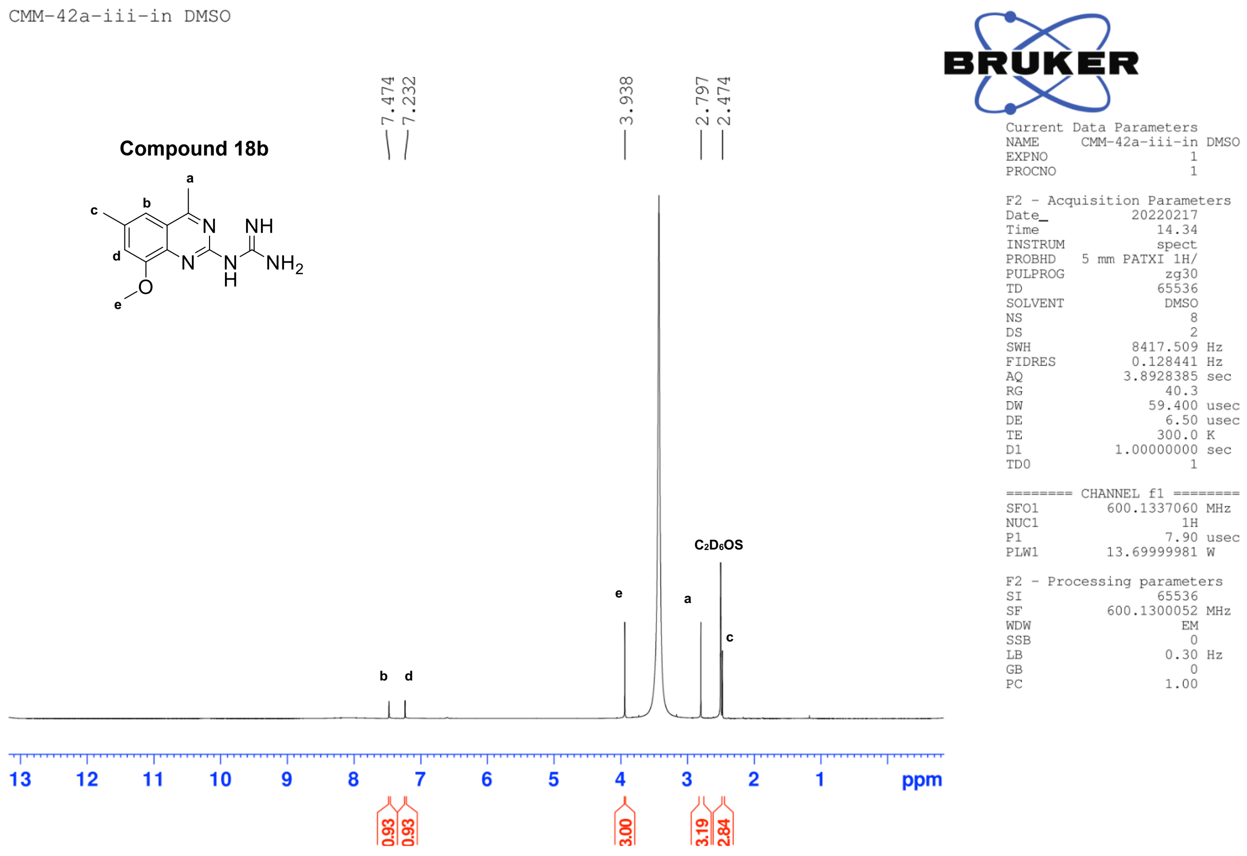

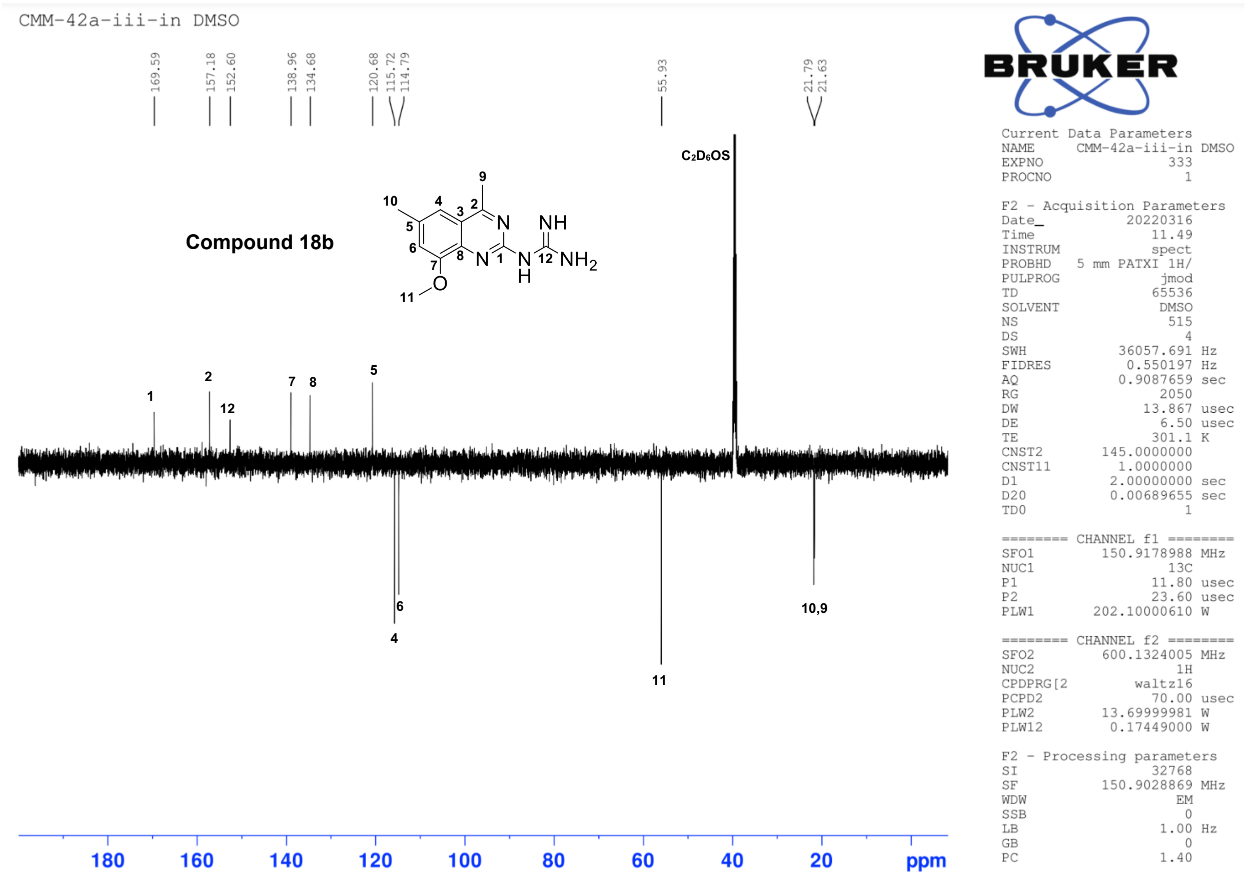

Compound 18b

LC-MS

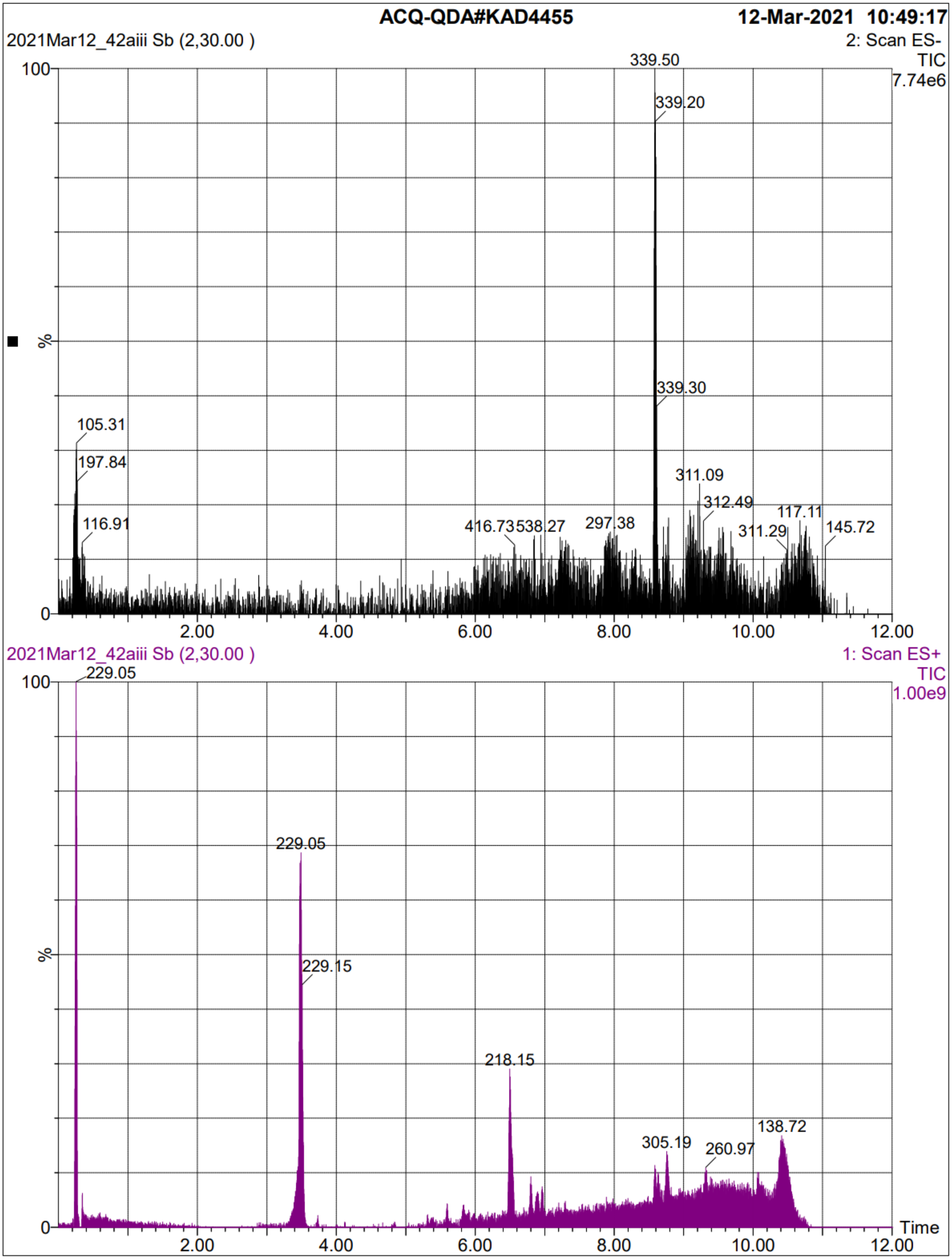

Compound 18b

MS

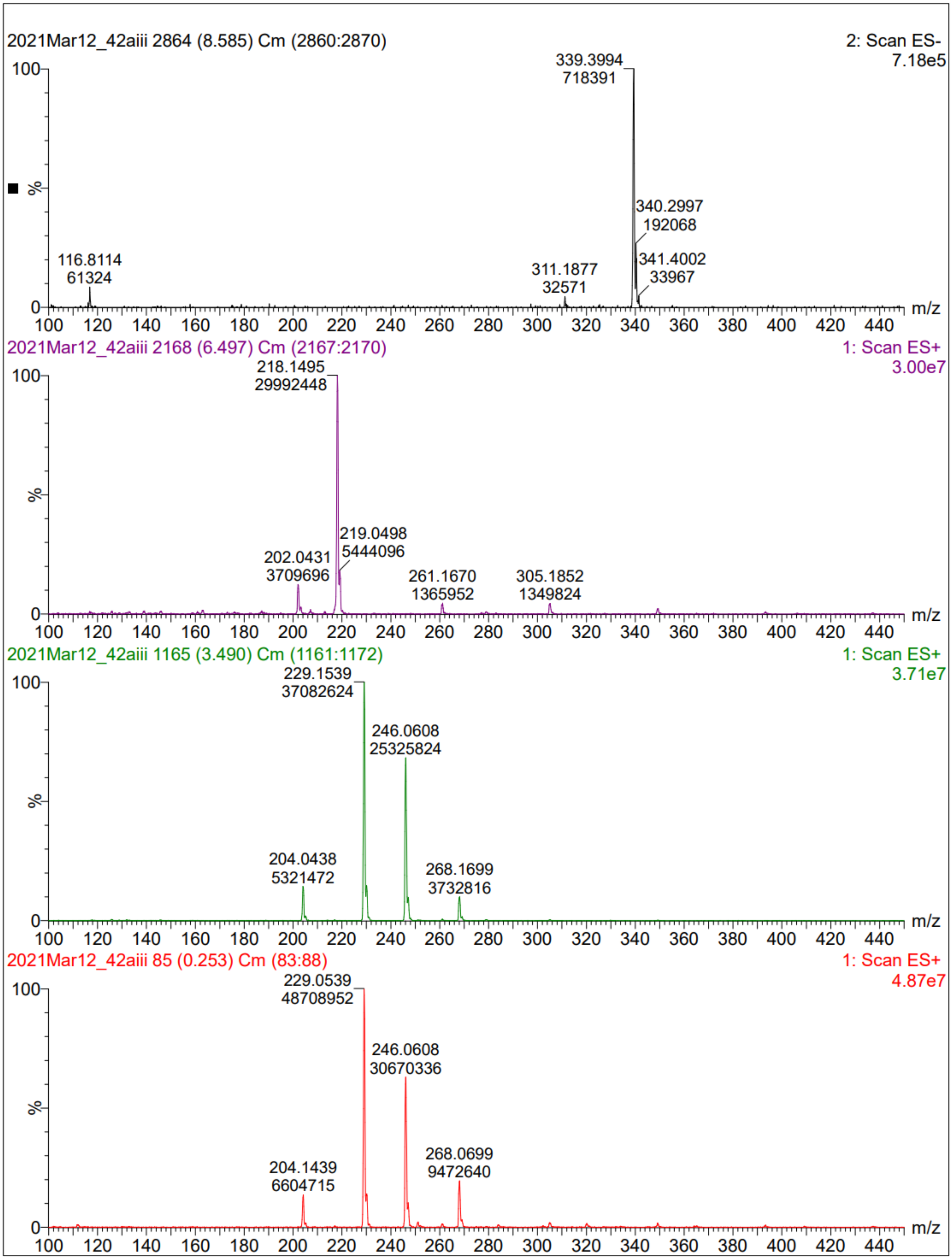

Compound 18b

Default Individual Report

SAMPLE INFORMATION

Sample Name:	42aii	Acquired By:	System
Sample Type:	Unknown	Sample Set Name	CMM210108_42 analogs_42aii
Vial:	1:A,7	Acq. Method Set:	Default
Injection #:	1	Processing Method	Default
Injection Volume:	5.00 ul	Channel Name:	PDA Max Plot 190.0 - 800.0
Run Time:	12.0 Minutes	Proc. Chnl. Descr.:	PDA MaxPlot (190.0 nm to 800.0
Date Acquired:	10/6/2022 9:51:55 AM EDT		
Date Processed:	10/6/2022 10:05:02 AM EDT		

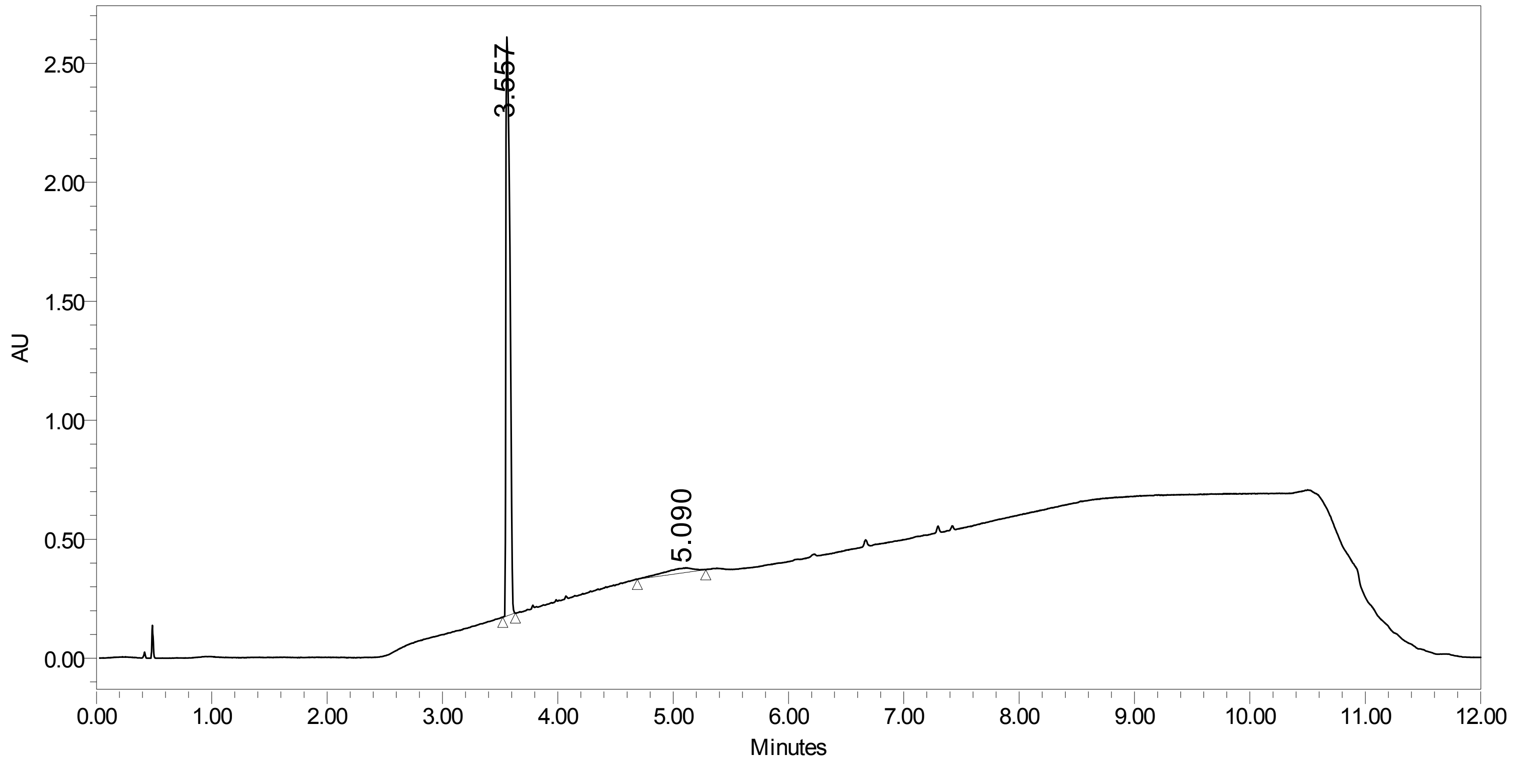

	RT	Area	% Area	Height
1	3.557	5878715	94.01	2434338
2	5.090	374643	5.99	20002

PDA Result Table

	Name	RT	Purity1 Angle	Purity1 Threshold	Match1 Spect. Name	Match1 Angle	Match1 Threshold
1		3.557	3.629	3.002			
2		5.090	24.589	33.076			

Compound **18c**

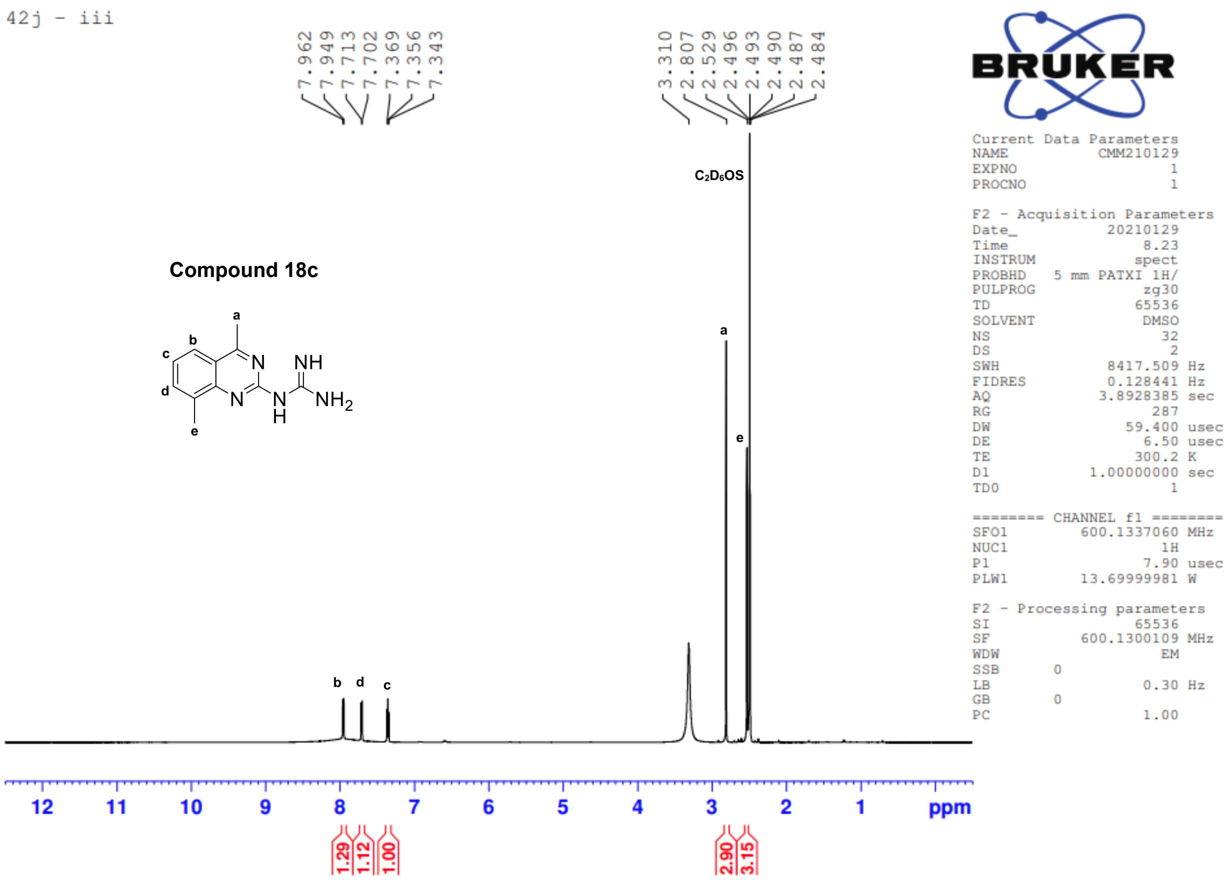

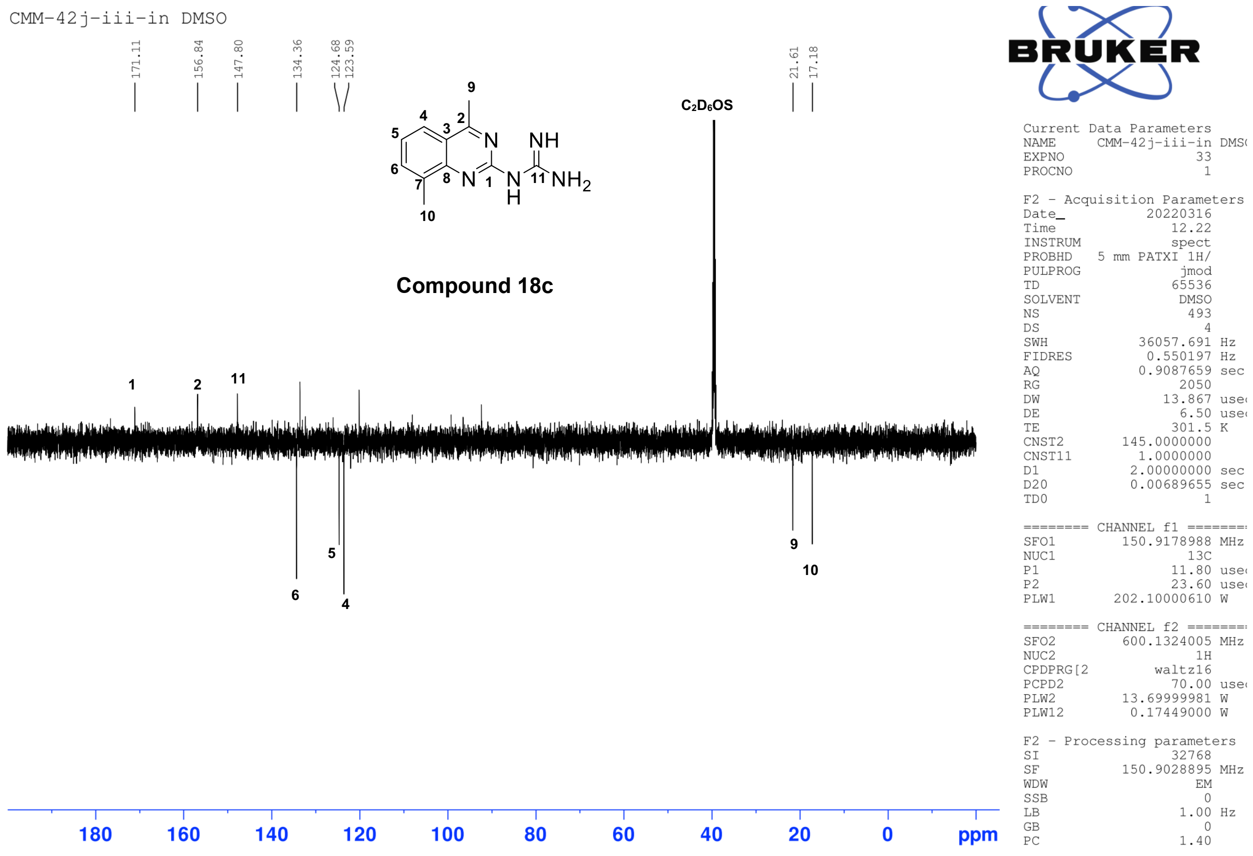

Compound 18c

LC-MS

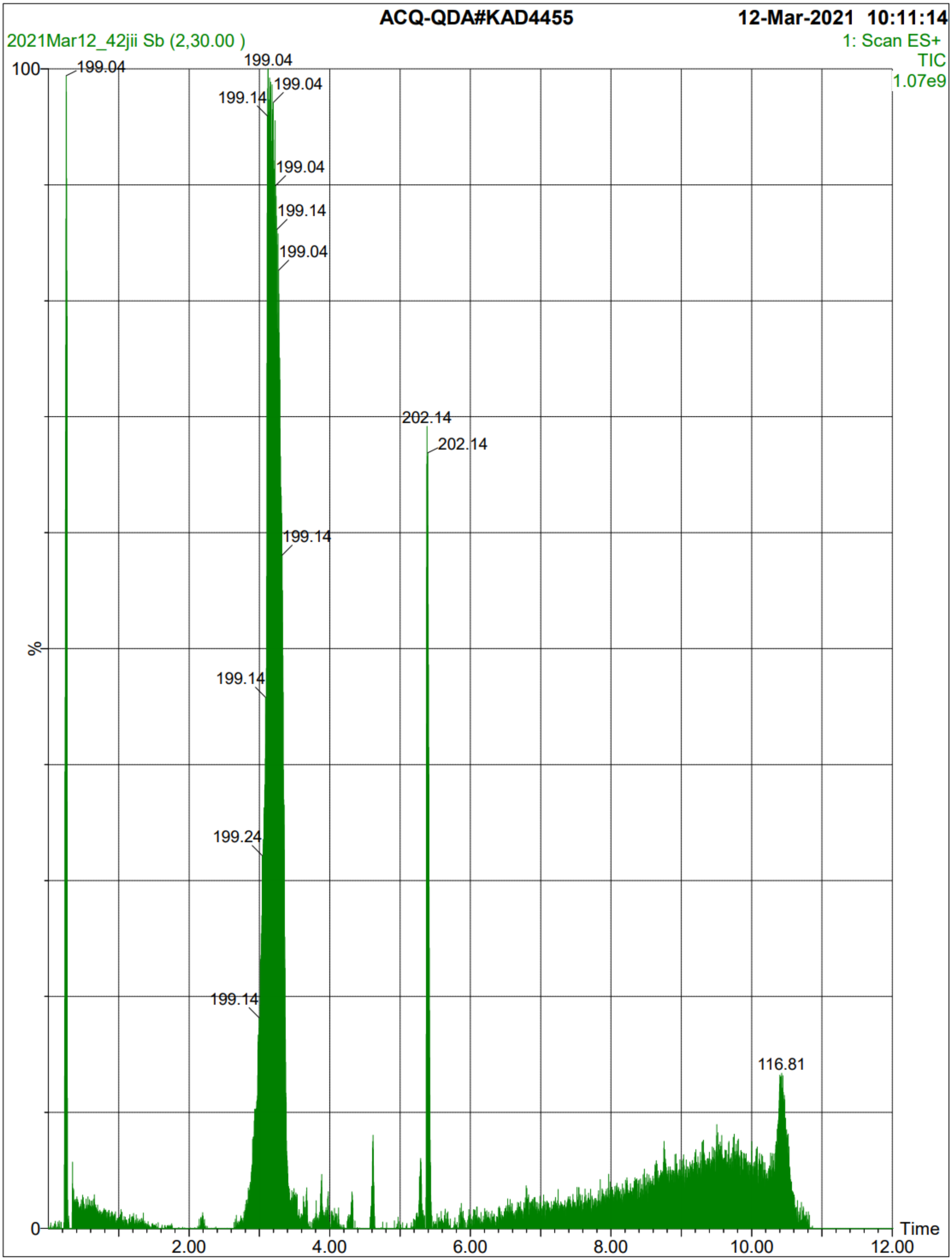

MS

Compound 18c

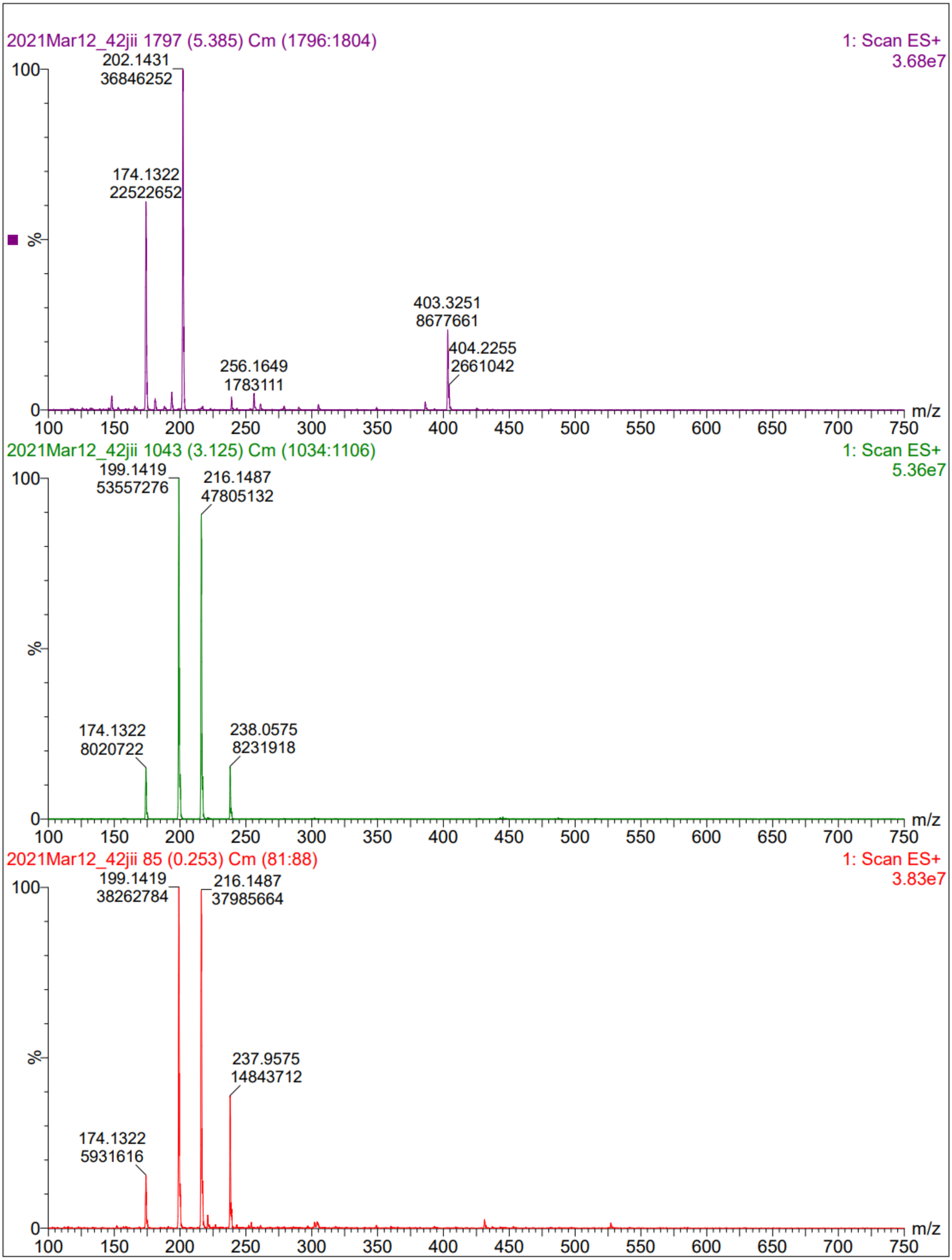

Empower 3 SOFTWARE

Compound 18c

Default Individual Report

SAMPLE INFORMATION

Sample Name:	42j_iii	Acquired By:	System
Sample Type:	Unknown	Sample Set Name	CMM210107_42 analogs
Vial:	1:A,5	Acq. Method Set:	Default
Injection #:	1	Processing Method	Default
Injection Volume:	5.00 ul	Channel Name:	PDA Max Plot 190.0 - 800.0
Run Time:	12.0 Minutes	Proc. Chnl. Descr.:	PDA MaxPlot (190.0 nm to 800.0
Date Acquired:	2/3/2021 12:01:30 PM EST		
Date Processed:	10/6/2022 8:15:52 AM EDT		

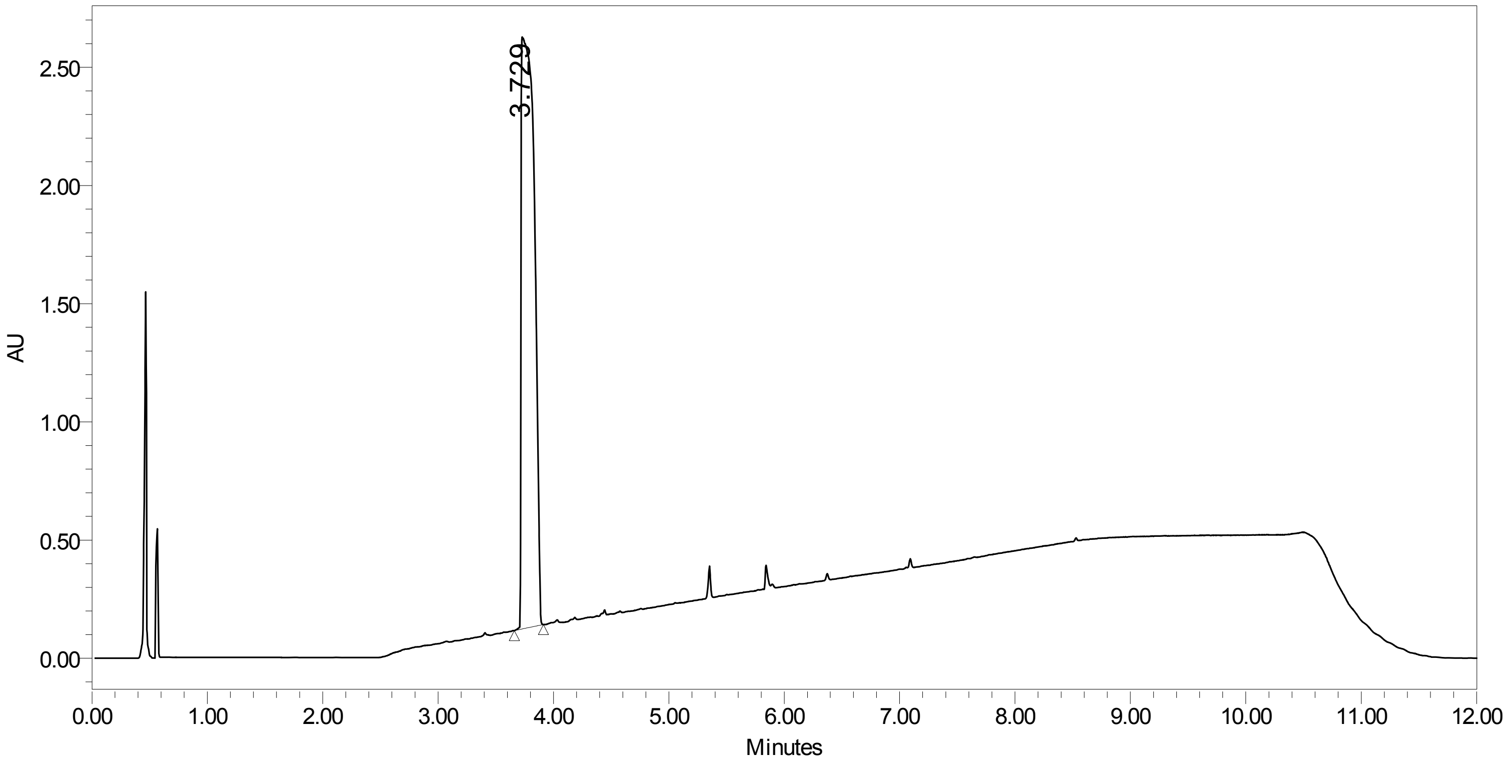

	RT	Area	% Area	Height
1	3.729	19637019	100.00	2504119

PDA Result Table

	Name	RT	Purity1 Angle	Purity1 Threshold	Match1 Spect. Name	Match1 Angle	Match1 Threshold
1		3.729	4.102	5.813			

Compound **2**

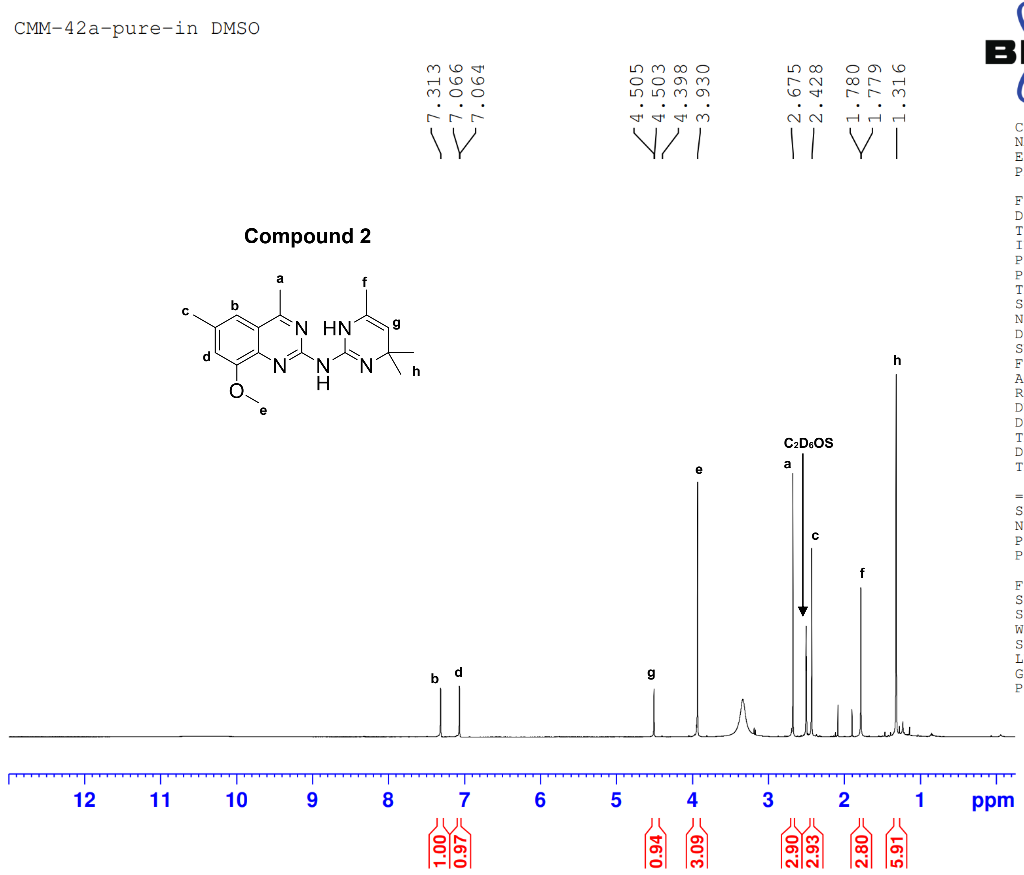


```
Current Data Parameters
NAME       CMM-42a-pure-in DMSO
EXPNO                      1
PROCNO                     1

F2 - Acquisition Parameters
Date_               20220218
Time                   10.18
INSTRUM                spect
PROBHD   5 mm PATXI 1H/
PULPROG                 zg30
TD                     65536
SOLVENT                 DMSO
NS                        16
DS                         2
SWH                 8417.509 Hz
FIDRES              0.128441 Hz
AQ                 3.8928385 sec
RG                       161
DW                    59.400 usec
DE                      6.50 usec
TE                     300.3 K
D1                1.00000000 sec
TD0                        1

======== CHANNEL f1 ========
SFO1          600.1337060 MHz
NUC1                      1H
P1                      7.90 usec
PLW1          13.69999981 W

F2 - Processing parameters
SI                     65536
SF            600.1300050 MHz
WDW                       EM
SSB        0
LB                      0.30 Hz
GB         0
PC                      1.00
```

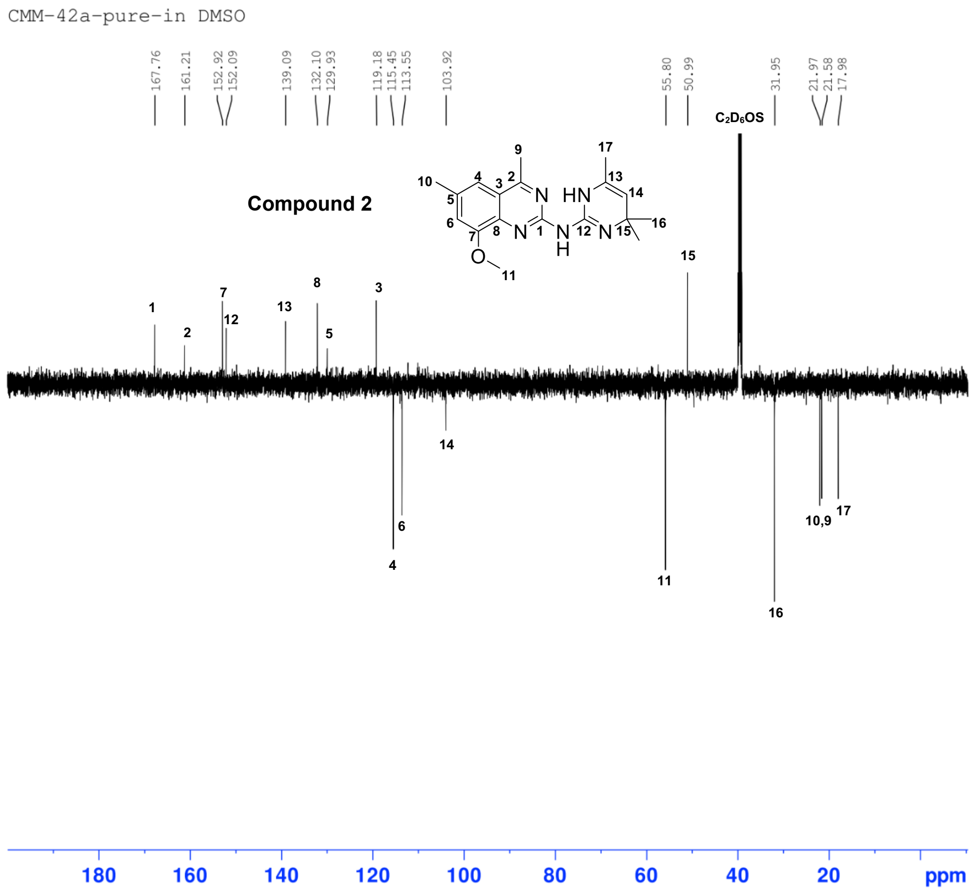


```
Current Data Parameters
NAME       CMM-42a-pure-in DMSO
EXPNO                      3
PROCNO                     1

F2 - Acquisition Parameters
Date_               20220218
Time                   11.31
INSTRUM                spect
PROBHD   5 mm PATXI 1H/
PULPROG                 jmod
TD                     65536
SOLVENT                 DMSO
NS                      1402
DS                         4
SWH                36057.691 Hz
FIDRES              0.550197 Hz
AQ                 0.9087659 sec
RG                      2050
DW                    13.867 usec
DE                      6.50 usec
TE                     301.4 K
CNST2            145.0000000
CNST11             1.0000000
D1                2.00000000 sec
D20               0.00689655 sec
TD0                        1

======== CHANNEL f1 ========
SFO1          150.9178988 MHz
NUC1                     13C
P1                     11.80 usec
P2                     23.60 usec
PLW1         202.10000610 W

======== CHANNEL f2 ========
SFO2          600.1324005 MHz
NUC2                      1H
CPDPRG[2             waltz16
PCPD2                  70.00 usec
PLW2          13.69999981 W
PLW12          0.17449000 W

F2 - Processing parameters
SI                     32768
SF            150.9028901 MHz
WDW                       EM
SSB                        0
LB                      1.00 Hz
GB                         0
PC                      1.40
```

Compound 2

LC-MS

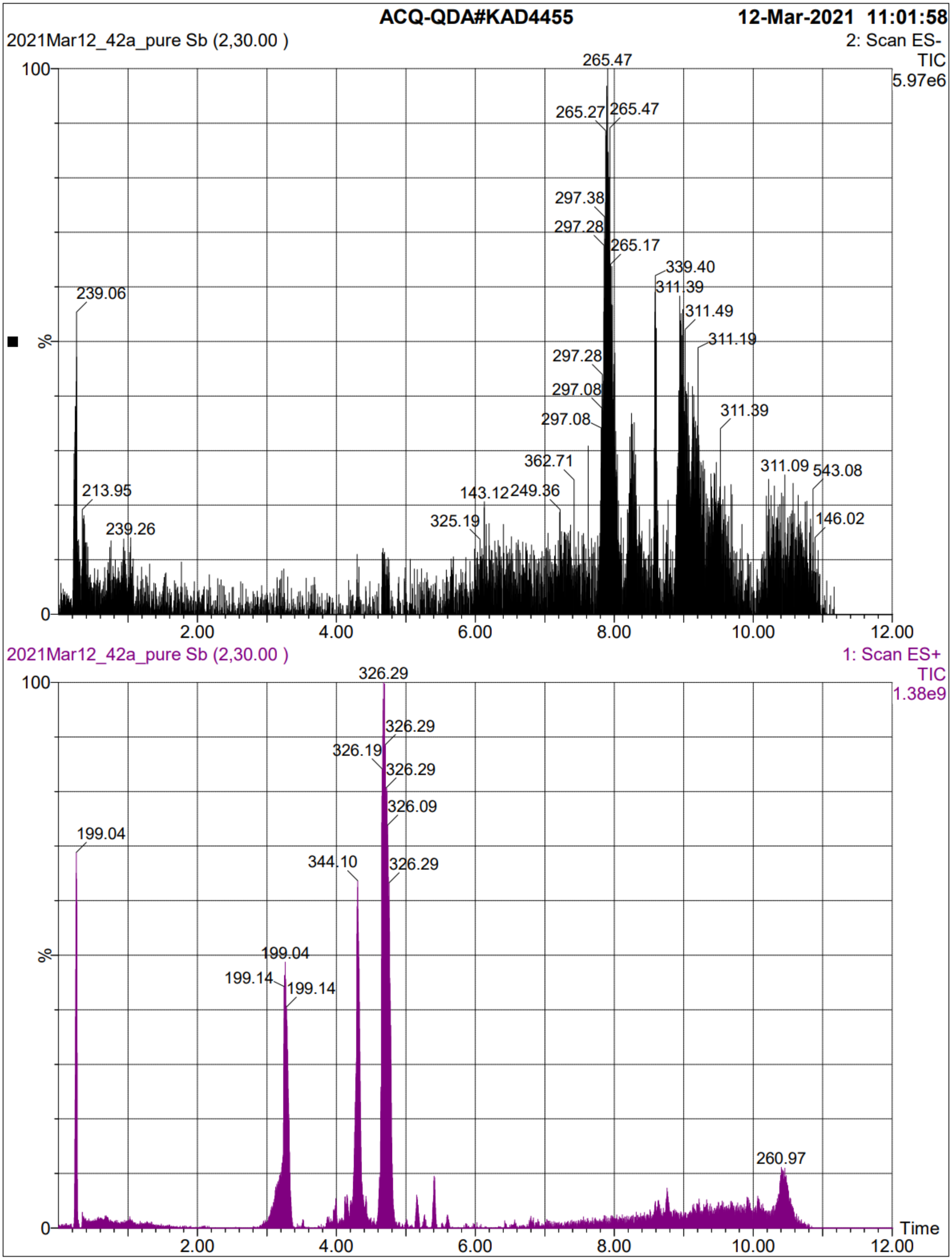

MS

Compound 2

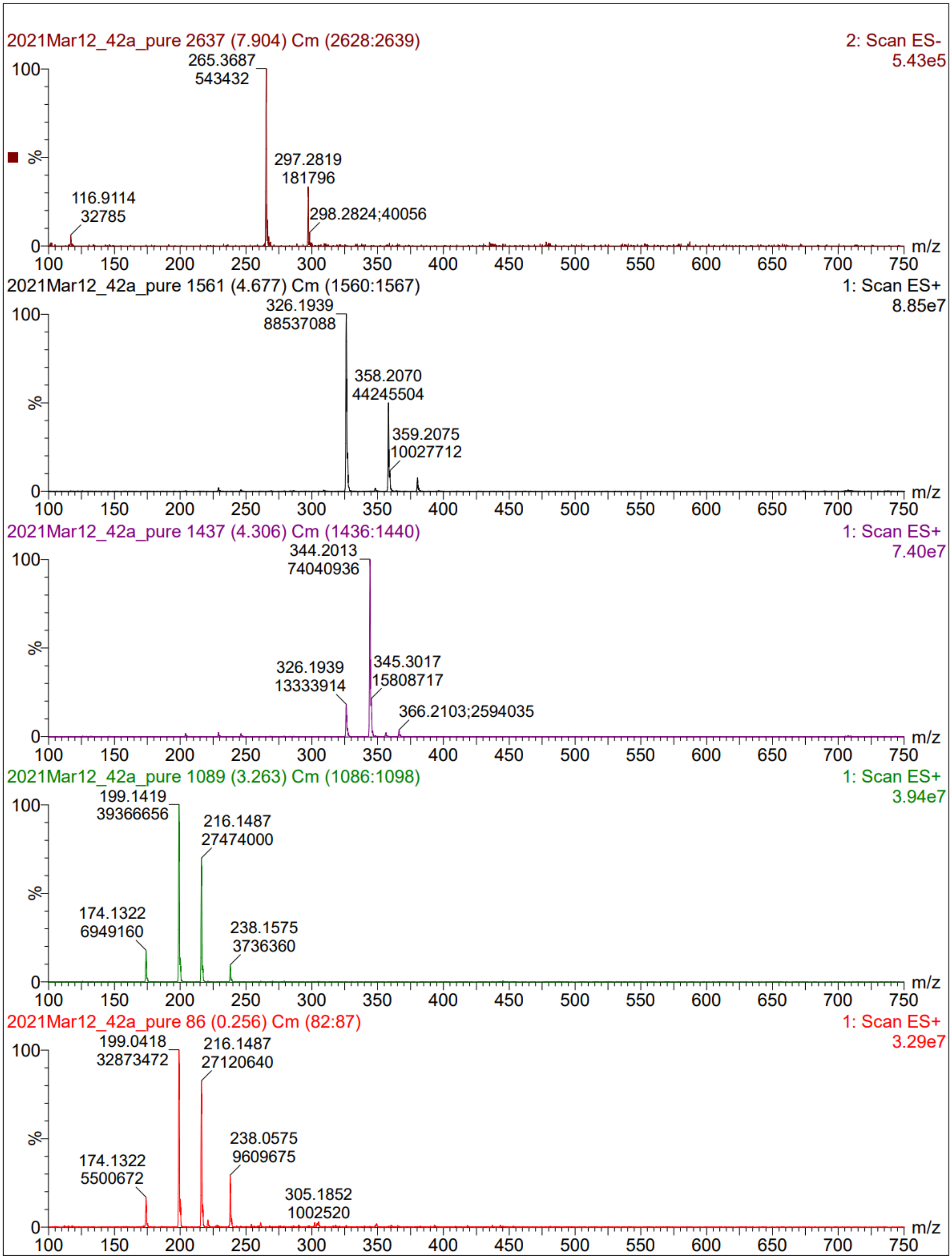

Compound 2

Default Individual Report

SAMPLE INFORMATION

Sample Name:	42a	Acquired By:	System
Sample Type:	Unknown	Sample Set Name	CMM210107_42 analogs
Vial:	1:A,4	Acq. Method Set:	Default
Injection #:	1	Processing Methoc	Default
Injection Volume:	5.00 ul	Channel Name:	PDA Max Plot 190.0 - 800.0
Run Time:	12.0 Minutes	Proc. Chnl. Descr.:	PDA MaxPlot (190.0 nm to 800.0
Date Acquired:	3/1/2021 2:11:46 PM EST		
Date Processed:	10/6/2022 8:06:48 AM EDT		

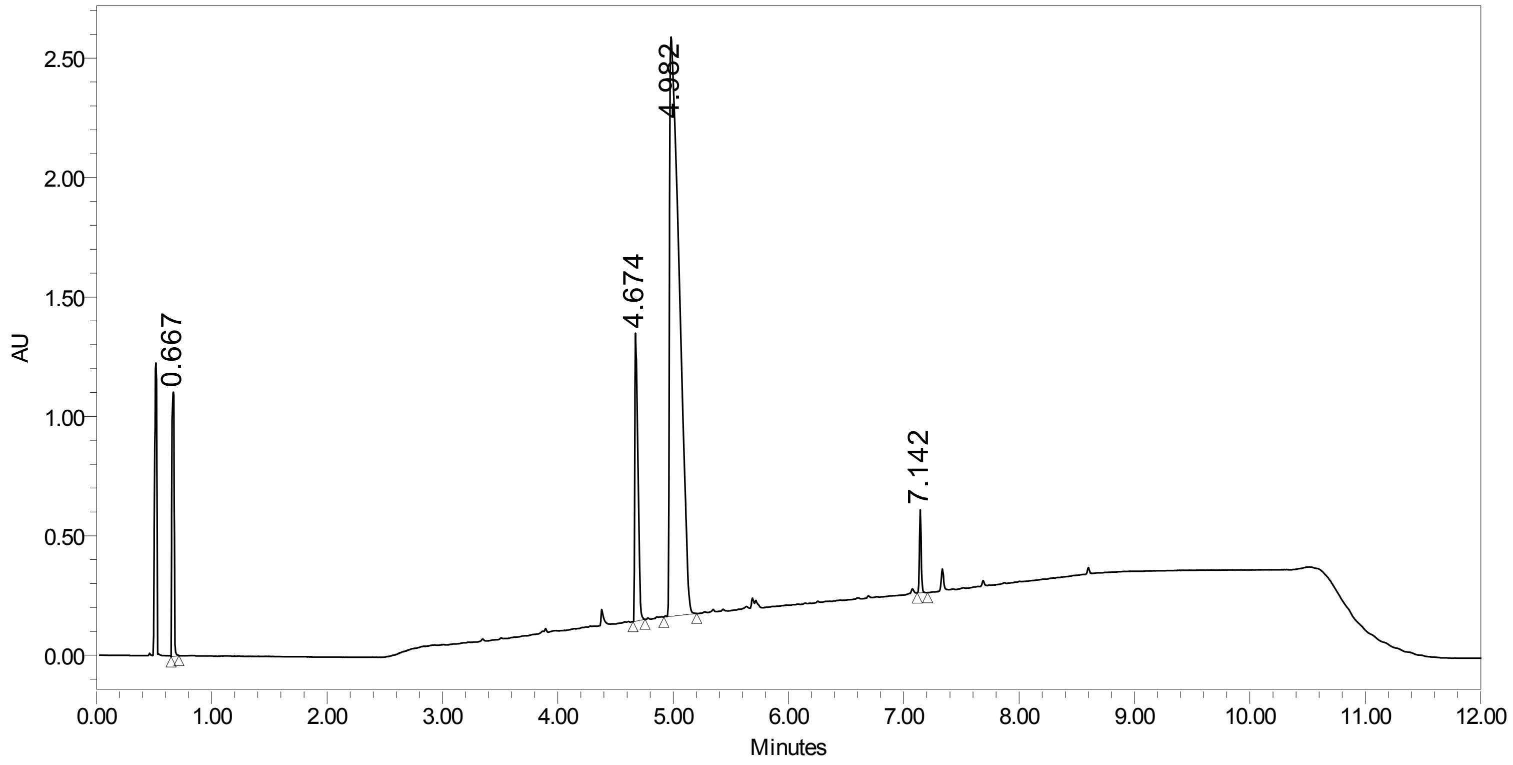

	RT	Area	% Area	Height
1	0.667	1591450	8.57	1107687
2	4.674	2308564	12.43	1206308
3	4.982	14318658	77.07	2426136
4	7.142	359628	1.94	348184

PDA Result Table

	Name	RT	Purity1 Angle	Purity1 Threshold	Match1 Spect. Name	Match1 Angle	Match1 Threshold
1		0.667	45.991	90.000			

PDA Result Table

	Name	RT	Purity1 Angle	Purity1 Threshold	Match1 Spect. Name	Match1 Angle	Match1 Threshold
2		4.674	1.834	1.373			
3		4.982	1.824	3.177			
4		7.142	13.590	9.673			

Compound **3**

CMM-42j-pure-in DMSO-220218

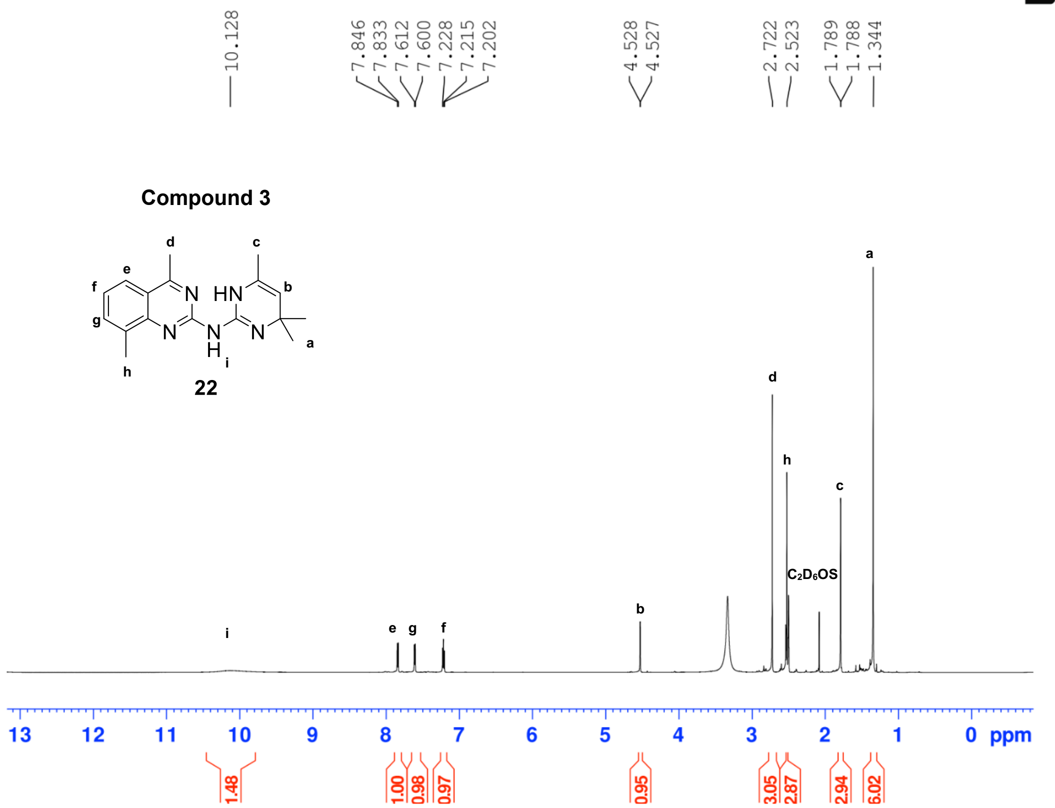


```
Current Data Parameters
NAME        CMM-42j-pure-in DMSO-220218
EXPNO                     11
PROCNO                     1

F2 - Acquisition Parameters
Date_               20220218
Time                   11.40
INSTRUM                spect
PROBHD    5 mm PATXI 1H/
PULPROG                 zg30
TD                     65536
SOLVENT                 DMSO
NS                         9
DS                         2
SWH                 8417.509 Hz
FIDRES              0.128441 Hz
AQ                 3.8928385 sec
RG                       114
DW                    59.400 usec
DE                      6.50 usec
TE                     300.6 K
D1                1.00000000 sec
TD0                        1

======== CHANNEL f1 ========
SFO1            600.1337060 MHz
NUC1                      1H
P1                      9.42 usec
PLW1             13.69999981 W

F2 - Processing parameters
SI                     65536
SF               600.1300050 MHz
WDW                       EM
SSB                        0
LB                      0.30 Hz
GB                         0
PC                      1.00
```

CMM-42j-pure-in DMSO-220218

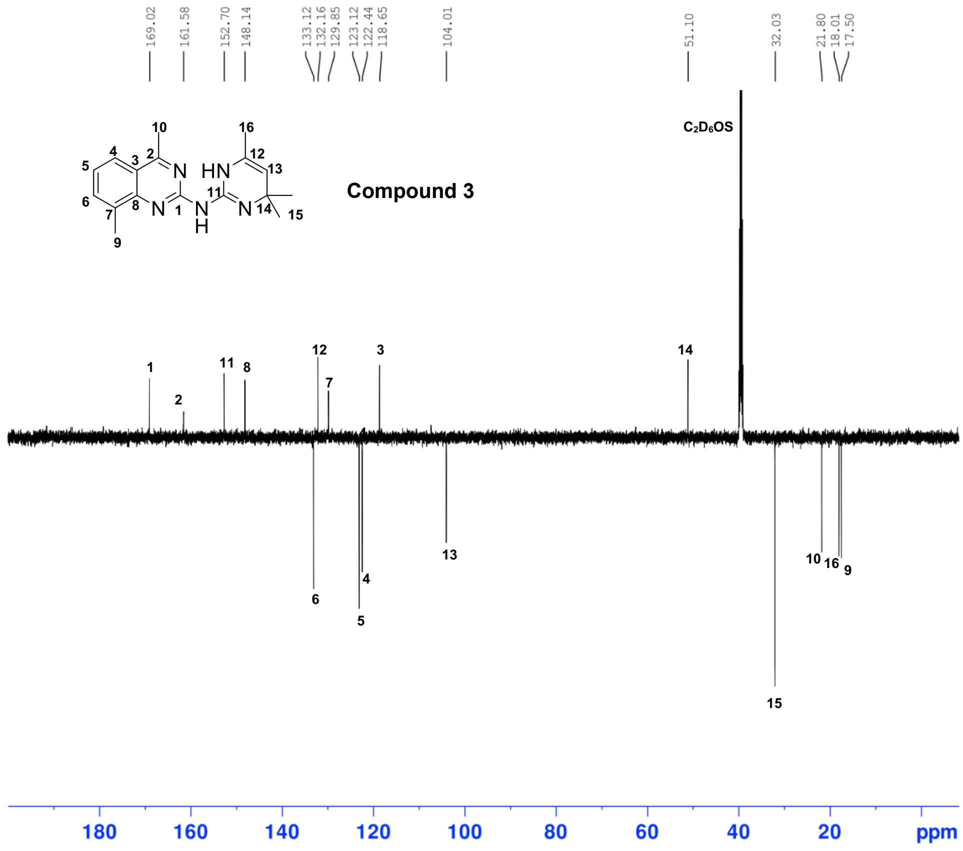


```
Current Data Parameters
NAME        CMM-42j-pure-in DMSO-220218
EXPNO                     33
PROCNO                     1

F2 - Acquisition Parameters
Date_               20220218
Time                   13.31
INSTRUM                spect
PROBHD    5 mm PATXI 1H/
PULPROG                 jmod
TD                     65536
SOLVENT                 DMSO
NS                      2224
DS                         4
SWH                36057.691 Hz
FIDRES              0.550197 Hz
AQ                 0.9087659 sec
RG                      2050
DW                    13.867 usec
DE                      6.50 usec
TE                     301.3 K
CNST2            145.0000000
CNST11             1.0000000
D1                2.00000000 sec
D20               0.00689655 sec
TD0                        1

======== CHANNEL f1 ========
SFO1            150.9178988 MHz
NUC1                     13C
P1                     11.80 usec
P2                     23.60 usec
PLW1            202.10000610 W

======== CHANNEL f2 ========
SFO2            600.1324005 MHz
NUC2                      1H
CPDPRG[2             waltz16
PCPD2                  70.00 usec
PLW2             13.69999981 W
PLW12             0.17449000 W

F2 - Processing parameters
SI                     32768
SF               150.9028907 MHz
WDW                       EM
SSB                        0
LB                      1.00 Hz
GB                         0
PC                      1.40
```

Compound 3

LC-MS

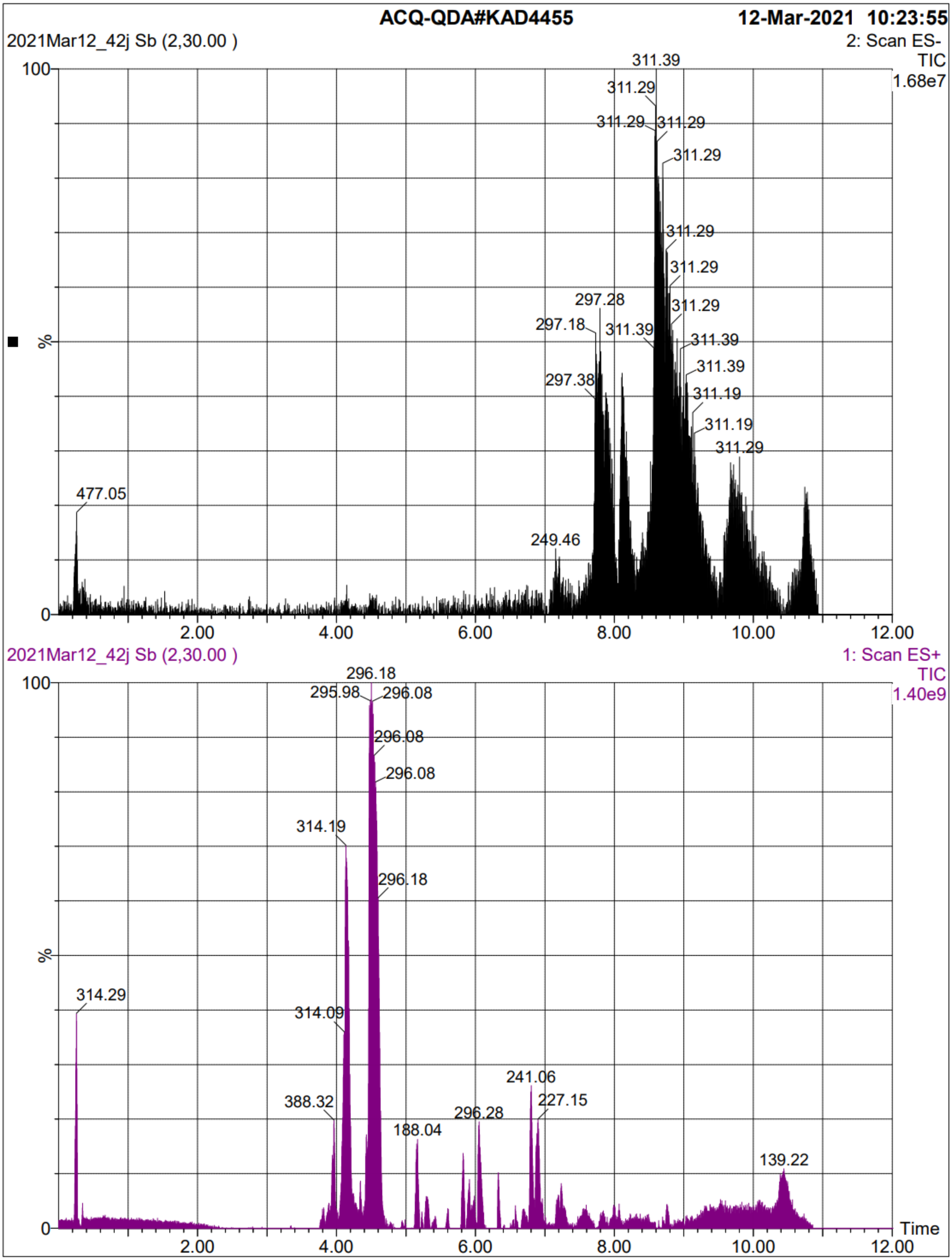

Compound 3

MS

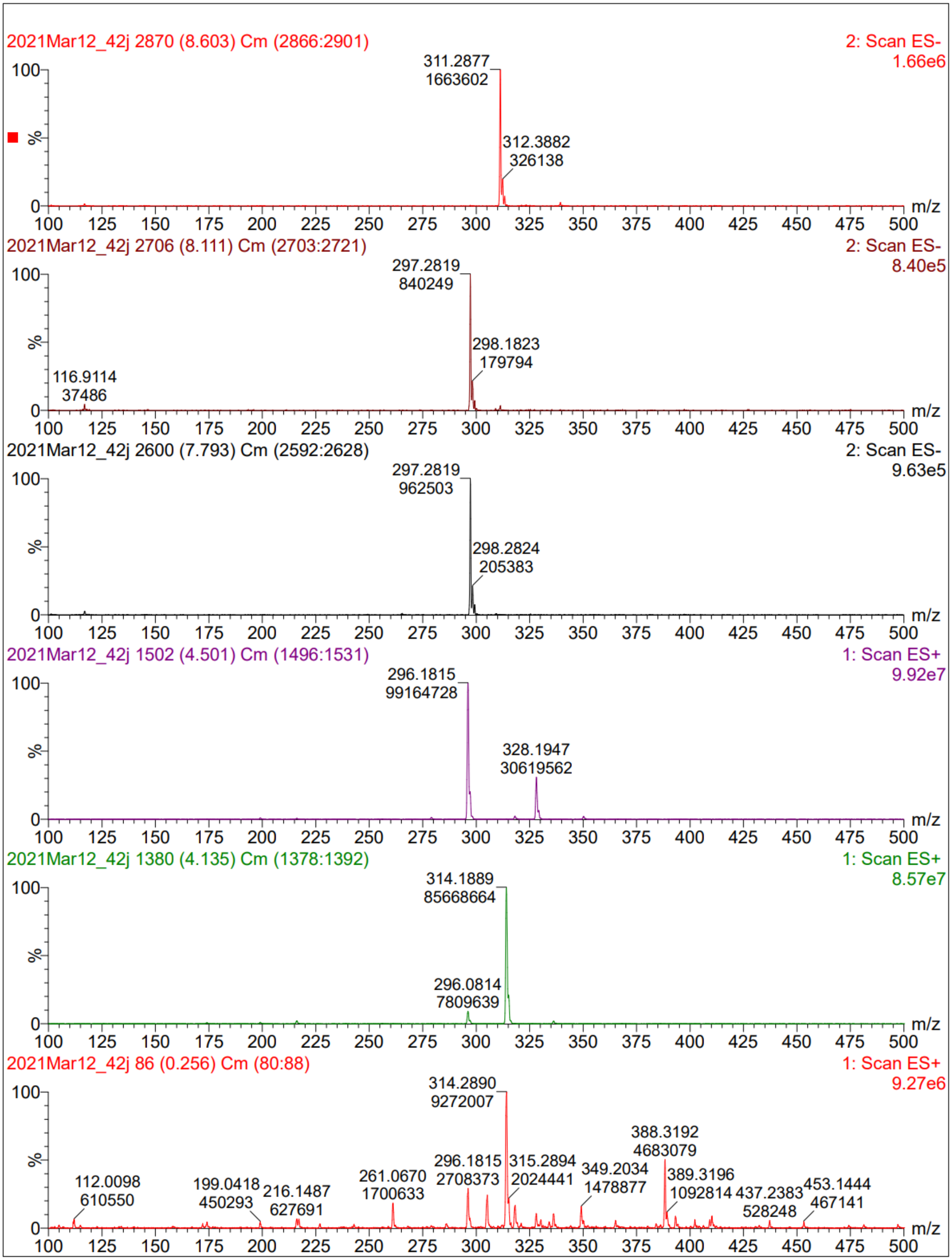

Compound 3

Default Individual Report

SAMPLE INFORMATION

Sample Name:	42j	Acquired By:	System
Sample Type:	Unknown	Sample Set Name	CMM210107_42 analogs
Vial:	1:A,4	Acq. Method Set:	Default
Injection #:	1	Processing Methoc	Default
Injection Volume:	5.00 ul	Channel Name:	PDA Max Plot 190.0 - 800.0
Run Time:	12.0 Minutes	Proc. Chnl. Descr.:	PDA MaxPlot (190.0 nm to 800.0
Date Acquired:	2/4/2021 11:26:35 AM EST		
Date Processed:	10/6/2022 8:10:59 AM EDT		

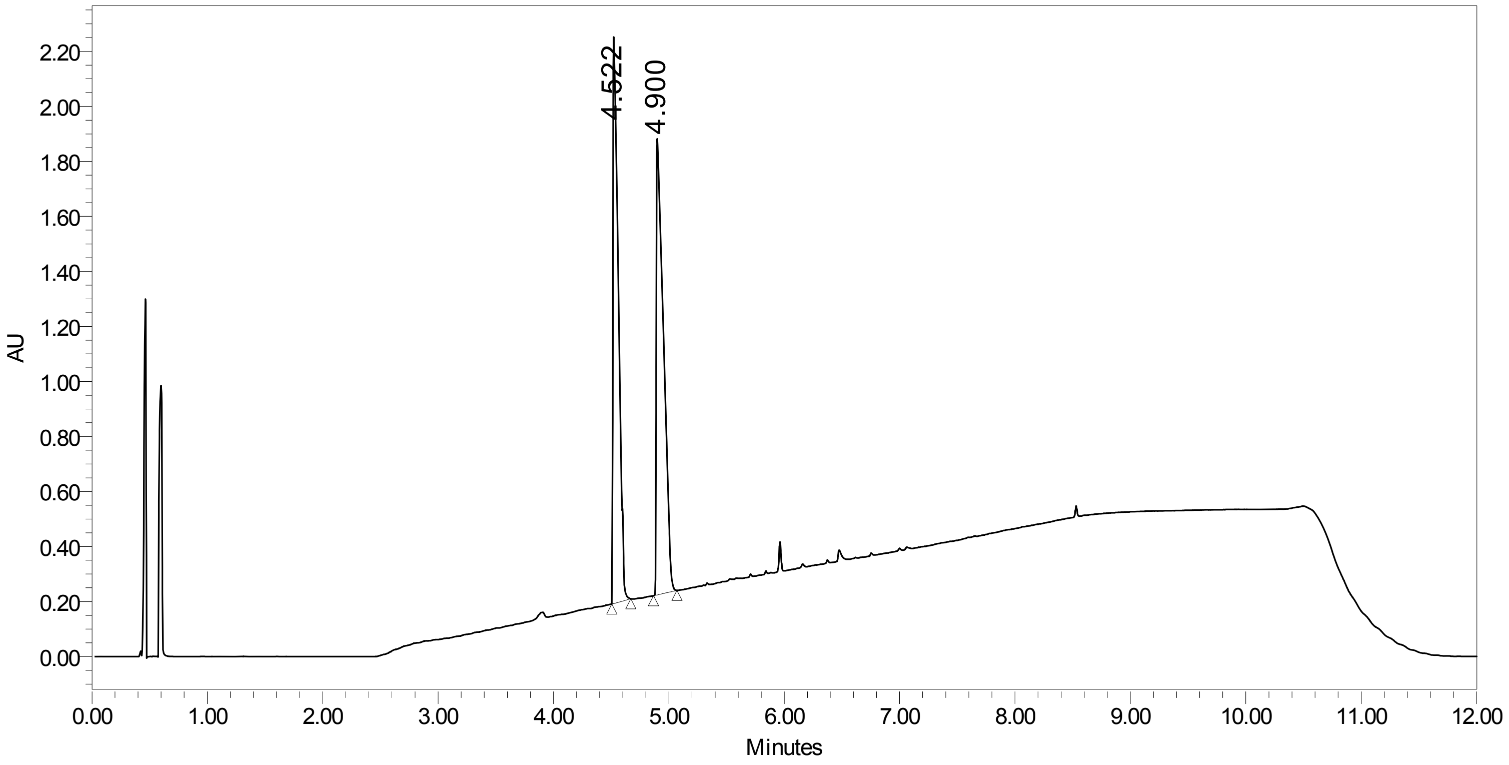

	RT	Area	% Area	Height
1	4.522	6715472	48.67	2060098
2	4.900	7081426	51.33	1658818

PDA Result Table

	Name	RT	Purity1 Angle	Purity1 Threshold	Match1 Spect. Name	Match1 Angle	Match1 Threshold
1		4.522	2.517	2.091			
2		4.900	1.135	1.025			
